# Brain–Computer Interface Spellers: A Review

**DOI:** 10.3390/brainsci8040057

**Published:** 2018-03-29

**Authors:** Aya Rezeika, Mihaly Benda, Piotr Stawicki, Felix Gembler, Abdul Saboor, Ivan Volosyak

**Affiliations:** Faculty of Technology and Bionics, Rhine-Waal University of Applied Sciences, 47533 Kleve, Germany; aya.rezeika@hochschule-rhein-waal.de (A.R.); mihaly.benda@hochschule-rhein-waal.de (M.B.); piotr.stawicki@hochschule-rhein-waal.de (P.S.); felix.gembler@hochschule-rhein-waal.de (F.G.); abdul.saboor@hochschule-rhein-waal.de (A.S.)

**Keywords:** Brain–Computer Interface (BCI), speller, Graphical User Interface (GUI), SSVEP, P300, MI, hybrid

## Abstract

A Brain–Computer Interface (BCI) provides a novel non-muscular communication method via brain signals. A BCI-speller can be considered as one of the first published BCI applications and has opened the gate for many advances in the field. Although many BCI-spellers have been developed during the last few decades, to our knowledge, no reviews have described the different spellers proposed and studied in this vital field. The presented speller systems are categorized according to major BCI paradigms: P300, steady-state visual evoked potential (SSVEP), and motor imagery (MI). Different BCI paradigms require specific electroencephalogram (EEG) signal features and lead to the development of appropriate Graphical User Interfaces (GUIs). The purpose of this review is to consolidate the most successful BCI-spellers published since 2010, while mentioning some other older systems which were built explicitly for spelling purposes. We aim to assist researchers and concerned individuals in the field by illustrating the highlights of different spellers and presenting them in one review. It is almost impossible to carry out an objective comparison between different spellers, as each has its variables, parameters, and conditions. However, the gathered information and the provided taxonomy about different BCI-spellers can be helpful, as it could identify suitable systems for first-hand users, as well as opportunities of development and learning from previous studies for BCI researchers.

## 1. Introduction

In this review, we primarily focus on the recent advances in the field of Brain–Computer Interface (BCI) spellers for different electroencephalogram (EEG) signals’ features. These speller systems are usually a graphical representation of letters, numbers, and symbols which are controlled using different BCI types for spelling and typing. Audio output can also be included by modern speech synthesis/voice recognition systems. 

The majority of research papers in the BCI field focus mainly on the development of the system’s back-end to improve the signal processing algorithms and boost the performance of the system (see the Research Methodology section). Our assumption is that, as the Graphical User Interface (GUI) of the BCI speller is the front-end, it is the first parameter which the end-user would judge on a BCI-speller, and, therefore, more attention should be given to it.

The goal of this paper is to describe and gather details about some unique and successful BCI-speller systems (from our point of view), specifically those published during this decade. The older state-of-the-art systems are discussed in this review as they are very well known and represent the basis on which many of the newer developments were built on. These systems were mainly developed with the objective of creating possible communication methods for patients suffering from motor neuron disease (MND) or with the goal of providing an initial proof of concept through examining the reliability of such systems by testing them with healthy subjects.

First of all, this review will benefit many researchers in the field as it provides a reference point giving an overview of the most successful BCI spelling systems. Consequently, it makes it easier and faster to go through many studies, facilitating the initial phase of a new research. Additionally, this review lists the improvements of different BCI-spellers, while highlighting the recent improvements and changes made with respect to the past and also presenting a taxonomy and classification of different features of such systems. This uncovers new development opportunities for further studies and underlines the relevant know-how for fresh researchers in the field. 

Another group which could benefit from this paper are the MND patients, their families, and caregivers, as they are the main targeted end-users for BCI spelling applications. As nowadays internet research is a common skill, many patients’ family members conduct internet lookups to find suitable rehabilitation systems or any new technology, which might help their afflicted relatives. Finding a review paper listing different options and developments of BCIs for communication (spelling) might be beneficial. It is difficult for healthy users and designers to anticipate the needs of an afflicted person. This review might encourage more patients to be willing to contribute with their opinions and testing for such systems. A BCI speller is characterized by features which attract the end-user. These characteristics are presented in an “easy-to-understand” taxonomy chart. End-users with basic or no prior understanding of the field could build a general knowledge about BCI spellers by reading through this review. The expectation is that there are potential end-users who are interested to learn about BCI. This review offers a smooth start from which MND patients (and patients with similar symptoms) with no previous knowledge or naïve understanding of BCI could begin improving their quality of life by using such systems for communication purposes. Publishing this review as an open access article might also reach more potential users and introduce them to BCI for the first time.

The review is structured as follows: Section 2 “Brain–Computer Interface” is a brief introduction and explanation of BCI in general, focusing on the relevant concepts in this review. Section 3, “Research Methodology”, describes the construction methodology of this review. Section 4, “Review of BCI Spellers”, presents the different types of BCI-speller GUIs, showing their features and characteristics with a short discussion concerning the described systems in each subsection. In Section 5 and Section 6, “Discussion” and “Conclusions”, a general discussion about BCI and similar systems is presented, while expressing our personal opinion about the upcoming development opportunities in the field.

## 2. Brain–Computer Interface

MNDs affect how the brain communicates with the other organs in the body by disrupting neurological networks; they mostly affect the motor control of the muscles. They include Amyotrophic Lateral Sclerosis (ALS). Similar symptoms are shown for Locked-in Syndrome (LIS), brainstem stroke, brain or spinal cord injury, cerebral palsy, muscular dystrophies, and multiple sclerosis, which eventually cause the afflicted patients to lose their ability to control voluntary muscles, mostly consisting of the skeletal muscles and the tongue, thus causing functional and cognitive disabilities. More details on MNDs can be found in [1]. As a result, these patients find it increasingly difficult to communicate with their surroundings, as they cannot speak or even use their hands for sign language. 

To help patients to regain their social life, an alternative way of communication is needed. An example of such communication systems, which has been around for years, are the eye-tracking spelling systems, which depend on the movement of the eye that controls a cursor on a virtual keyboard and selects the desired letters [2]. Also, a simple eye blinking can be used as a communication method. Such and similar systems might not be suitable for some patients who have lost the ability to precisely control fine ocular movements or who experience uncontrollable head movements [1,3]. 

A solution which would allow these patients to communicate is the utilization of modern BCI. A BCI system allows people to communicate through brain signals without the need of any muscular movement. It provides an artificial output that is different from the usual natural output of the nervous system, which is disabled in most MND patients. 

There are many methods to monitor the brain’s activity. One of the most common methods for measuring brain waves, and our focus during this review, is the electroencephalogram (EEG) [4]. EEG is a non-invasive measurement technique widely used in almost all modern BCI applications, more practical than Electrocorticography (ECoG), which requires an opening through the skull to directly access the brain tissue [4]. The main reasons why EEG is so common are: EEG equipment is relatively inexpensive, portable, simple to set-up, and provides a signal with high time resolution compared to other non-invasive methods for monitoring brain activity like Magnetic Resonance Imaging (MRI) or Positron Emission Tomography (PET), just to mention a few [5]. Also, non-invasive BCIs could become a useful tool to be utilized and tested by healthy individuals for research and development of applications [4]. 

After measuring and recording the brain activity in a BCI system, specific features of the signal are extracted and analyzed by the computer. This output has the potential to serve as a BCI application which might replace, restore, enhance, supplement, or improve the function of the central nervous system [6]. 

Over the recent years, BCI researchers have been developing various applications which might be useful for MND patients in particular. One of the most commonly studied applications is the BCI-speller. Usually, a BCI-spelling application allows the users to communicate with environment using a GUI. The GUI displays letters, numbers, and special characters. With the aid of the brain signal recorded and analyzed by the BCI system, the user selects the desired character and types it on the screen or other output displays. Farwell and Donchin presented the first spelling application in 1988 [7]. Promising accuracy levels and typing speeds have been presented in the literature since then. Consequently, BCI spelling applications were further developed, allowing people to communicate directly through the measurements and direct interpretation of brain activities. In general, the loss of communication for such patients affects their quality of life negatively as presented in [8]. Subjects using a BCI-speller can be more independent and can even regain their social life to a relatively high extent. 

The measured brain activity from the BCI is interpreted with the intention of selecting the desired key (letter, number, or symbol) shown on the screen. In contrast to standard physical keyboards used traditionally in most computer systems, where the user selects the desired key by physically pressing it, in a BCI system, the user selects a key by looking at it (or by other sensory modalities in some cases), and the letter will be “pressed” by the computer according to the measured and classified brain signals. 

The performance of BCI-spellers is commonly measured by calculating the accuracy and the Information Transfer Rate (ITR) of the system. The accuracy is calculated by dividing the number of correct commands by the total number of commands. The commonly used ITR was introduced by Wolpaw in [9], originally presented much earlier, as discussed in [10]. The ITR combines the accuracy and the system’s speed in one variable and it is expressed as the number of error-free bits per time unit. It is important to note that the ITR may be calculated in different ways (e.g., on the level of commands or of the letters) in different types of BCIs. It can only be used objectively to compare the performances of systems of the same type.

This review focuses on the main EEG paradigms used by the vast majority of BCI spellers: Event-Related Potentials (ERP) (mainly P300 and Steady-State Evoked Potential (SSEP)) and motor imagery (MI, also called Event-Related Desynchronization/Synchronization (ERD/ERS)) [4,5]. 

### 2.1. Event-Related Potential (ERP)

ERPs are electrocortical signals which can be detected and measured using EEG, during or after a sensory, motor, or psychological event. They usually have a fixed known time delay to a stimulus and a different amplitude compared to the spontaneous EEG activity. ERPs are less frequent and more localized than the normal EEG-measured signal. Different ERPs can be evoked using different types of stimulus (events), and the evoked ERP is characterized by a specific time delay and/or location where it was generated. The two most common ERPs are the P300 and the Steady-State Visual Evoked Potential [11].

The P300 wave is a type of event-related potential which occurs in the human brain as a positive deflection with a time delay of around 300 ms after a specific event has occurred [12] (although the timings may vary, as discussed in [13]). The P300 signal is usually intensified over the central parietal region of the brain and can be detected using EEG. The event which stimulates the P300 is known as “the oddball paradigm” [7]. Accordingly, this paradigm consists of three main prerequisites [6]:

- A subject is presented with a series of stimuli or events; each of them belongs to one of two classes (e.g., a desired or an undesired event)
- One of the classes is less frequently presented than the other class (a rare event versus a usual event)
- The subject needs to pay attention to one of the stimuli when it occurs (e.g., counting how many times a particular letter will flash, which is the rare event).

**Figure 1:**
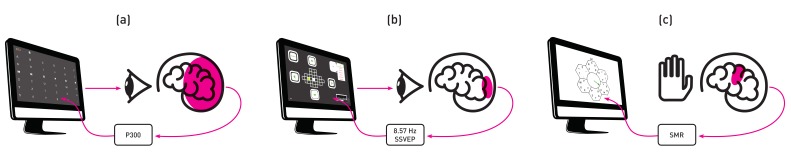
Schematic representation of three major Brain–Computer Interface (BCI) paradigms: (**a**) P300 paradigm. The oddball paradigm causes a P300 signal in the brain of the user which is then interpreted by the BCI system, resulting in the selection of the desired letter; (**b**) Steady-State Visual Evoked Potential (SSVEP) paradigm. Five different frequencies are shown on the screen in this example, as discussed later. When the user gazes at one of them, an SSVEP signal with the same frequency (as well as its harmonics) is elicited in the visual cortex of the brain. The measured electroencephalogram (EEG) data are analyzed by the BCI, and a command is sent to the computer to select the target; (**c**) Motor imagery (MI) paradigm (with a schematic representation of a Hex-O-Spell application, as discussed later). The imagination of the movement of limbs (in this picture an imaginary movement of an arm) induces a sensorimotor rhythm (SMR) signal which is detected and analyzed by the BCI system, and a feedback is sent to the computer to control the movement of the green arrow for letter selection. In this case, the presence of an external stimulus is not required.

The rare events induce the P300 signal in the brain. Researchers have developed both visual and auditory stimuli to induce a P300 signal for different systems and applications (Visual Evoked Potential (VEP) and Auditory Evoked Potential (AEP)). One of the first BCIs using the P300 signal is a speller developed by Farwell and Donchin in 1988 (Figure 1a) [7], which used visual stimulation for the “the oddball paradigm”. Hill et al. in 2005 [14] introduced the first P300 BCI based on auditory stimuli. In 2014, a novel auditory speller, named “charstreamer”, was presented by Höhne et al. [15]. As for other types of sensory stimulation, in [16], a tactile P300 BCI was developed by fixing vibration motors at different locations around the participant’s waist. The user had to focus on the vibration at the desired location and ignore all the others to elicit a P300 signal. More recently, researchers started experimenting with placing motors on different parts of the body, such as the back or the hand of the user, with the aim to improve the tactile P300 BCI performance [16,17]. 

The Steady-State Evoked Potential (SSEP), specifically the Steady-State Visual Evoked Potential (a type of VEP) (Figure 1b), is characterized by positive and negative fluctuations in the EEG signal which are responses to a visual stimulus. For example, light is flashing, an image is appearing/disappearing, or a pattern is presented with a certain frequency. SSVEP is recognizable in the EEG recordings as voltage oscillations which are further processed to detect their unique features, such as frequency and amplitude. When the external visual stimulus is flickering at a specific constant frequency, an SSVEP is elicited with a peak frequency matching the stimulus (as well as its harmonics), mainly in the visual cortex, located in the occipital region of the brain, given that the subject’s eyes are fixated on the stimulus. Usually, a frequency analysis technique, such as Fast Fourier Transform (FFT), is used to detect the stimulation frequency [6]. 

In a standard SSVEP system, taking a spelling application as an example, the targets can be individual letters or groups of characters or command boxes. Each target flickers with a unique frequency. This is also known as frequency-modulated Visual Evoked Potential (f-VEP). Another well-known type of VEP is the so-called code-modulated Visual Evoked Potential (c-VEP). Instead of using a constant flickering frequency, the stimulus is a pseudorandom swapping of orthogonal patterns [18]. It is worth adding that tactile stimuli were also used to elicit an SSEP response in a BCI system [19]. 

Another type of VEP is the Motion-Onset Visual Evoked Potential (mVEP). The above mentioned VEPs depend mainly on light flashes or patterns. In 2008, Fei Guo et al. [20] presented a different approach, the first BCI system based on mVEP. In [20], visual responses from the dorsal pathway of the visual system were utilized, which led to the use of more elegant visual stimuli. The mVEP paradigm has been used for several years to investigate human brain motion processing [21]. It is typically comprised of three main peaks: P1, N2, and P2. The N2 peak, with a latency of 160–200 ms, is predominantly motion-specific, and the P2, with a delay of about 240 ms, is elicited with more complex visual moving stimuli [22,23,24]. The mVEPs are usually elicited by a pre-defined simple motion of the visual targets. 

### 2.2. Movement Imagination 

The sensorimotor rhythm (SMR) (Figure 1c) can be recorded over the motor cortex with the contribution of some somatosensory areas. During movement, Motor Imagery (MI) and movement preparation the SMR can be decreased or increased; these options are known as Event-Related Desynchronization (ERD) and Event-Related Synchronization (ERS), respectively [11]. During ERD, the signal drifts and becomes lower than a specific baseline, which might be due to the desynchronization of the activities of specific areas of the brain [11]. On the other hand, during ERS, the signal measured during movement is stronger when compared to a baseline. The signal location varies depending on which limb is moving and on which side of the body the specific movement is taking place. It was also discovered that the imagination of a movement without actually performing it elicits a similar EEG signal [25]. Even though this signal is weak in comparison to ERP and VEP, leg and arm movements can be distinguished, as well as the side of the upper limb (left or right) [6].

## 3. Research Methodology

**Figure 2:**
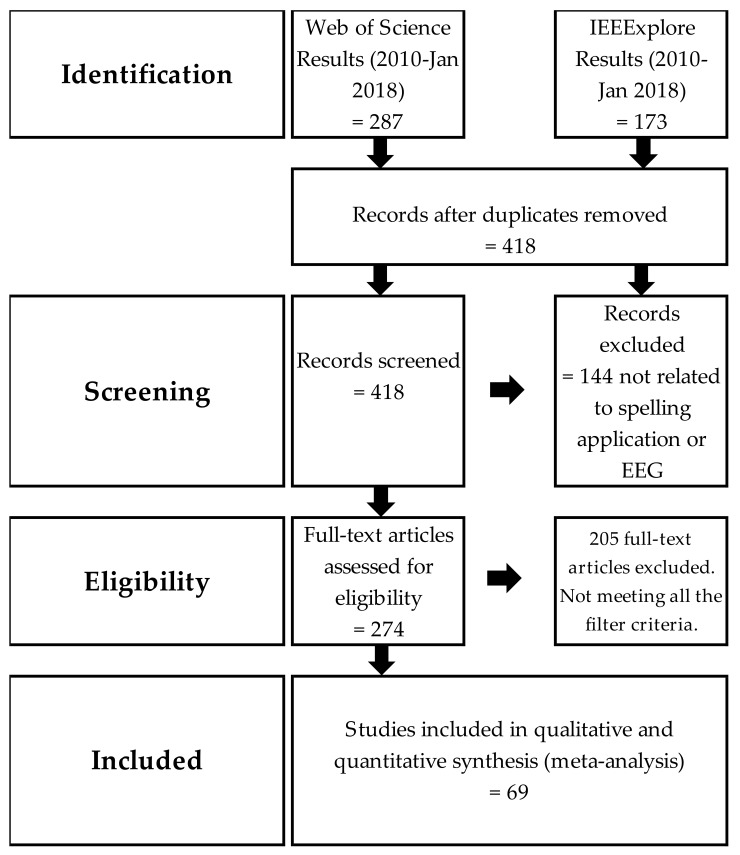
PRISMA chart.

Literature research was conducted according to the PRISMA guidelines [26] (PRISMA diagram shown in Figure 2), using the IEEEXplore Digital Library (incl. conference proceedings) and further online databases through Web of Science (WOS). In both, the search was conducted using the search terms “BCI” AND “speller”, and the dates were restricted from 2010 to January 2018. First, the search was done without constraining the years, as a test. WOS showed 316 results, and IEEE showed 213 results, giving a total of 529 (including duplicates if any). Later, the search was performed with time restriction. WOS showed 287 results, and IEEE showed 173 results, giving a total of 460. From this observation, we deduced that the steep growth of research during this decade deserved a deeper look, taking into account the origin of these developments from earlier years.

Both lists (WOS time-restricted and IEEE time-restricted) were extracted for analysis. Duplicates were detected, as some of the IEEE conference proceedings and journal papers were listed on the WOS database. First, the duplicates were removed, resulting in a total of 412 papers and articles. Then, the papers were checked and classified manually according to BCI type, output type, number of subjects, type of subjects, and purpose of the research. The final step was to determine which papers were relevant to the topic of our review. The filtration of 412 remarkable papers and articles according to the below-mentioned classification criteria used in this review was a delicate process, which took an extensive number of working hours:

- Non-invasive BCI = EEG-based BCIs
- Only visual stimuli or movement imagery
- The purpose of the research or the aim of the research is the development of a new Graphical User Interface (GUI) for a BCI speller system OR a clear modification of an existing GUI
- Published between 2010 and January 2018 (with few exceptions).

### BCI Spellers Taxonomy

All BCI speller systems can also be categorized according to the following characteristics: dependent or independent, synchronous or asynchronous, with regard to the stimulus type and gaze dependency. 

The P300 and SSVEP depend on visual stimulation to induce a specific brain activity which can be later interpreted by the BCI system. Thus, a stimulus must be physically present in the environment to initiate the required signal. The P300 and VEP require a structured environment to present external stimuli. Such BCIs are usually implemented as dependent BCI. The MI-BCI, however, depends on the imagination of the movement of any limb, whole-body activities, performing of specific cognitive tasks, relaxation, etc. This imagination initiates a brain activity in the motor cortex region of the brain, which can be detected and interpreted by the BCI system. In this case, only the subject is responsible for creating and firing up the desired brain signal; MI-BCI systems are therefore classified as independent BCIs, as no external (e.g., visual) stimuli are required. 

A synchronous system limits the time intervals when the BCI will process the measured and analyzed brain activity into actions [27]. It provides a starting point and measures a specific brain signal which happens afterward. Thus, the system limits and specifies the time at which the BCI can use the measured activity to produce a useful output. In the meantime, the protocol might provide the user with cues to alert the user to get ready and prepare for the coming stimulation phase. Usually, synchronous BCIs do not consider the possibility that, at a specific point in time, the user has no intent to use the system. The commonly used P300 speller application based on [7] is a typical dependent synchronous BCI. The P300 stimuli occur for a specific, pre-defined period in which the user has to focus the attention on the displayed GUI for a meaningful output. In addition, some MI applications specify the time slot where the user should imagine the movement, i.e., the user has to wait for a cue to perform a movement imagination, otherwise an error would occur [6]. 

The asynchronous (or self-paced) protocol is simply the opposite. The user has the ultimate control over the system, whenever they desire. Asynchronous BCIs result in a more natural and dynamic interaction between the user and the system. The user does not have to wait for a cue to control the system. The SSVEP-based BCI system can also be created as an asynchronous BCI, e.g., the user directs his/her focus of attention on the flickering SSVEP stimuli (when the user shifts her/his gaze away from the stimuli = no classification) [6].

Moreover, recent studies are investigating BCI-spellers according to the type of attention needed, whether it is overt or covert. Overt attention occurs when eye movement is involved in paying attention to a specific visual space or region. Covert attention is more of a mental attention and not a specific visual attention. While the eyes are fixed, the attention is shifted mentally to the desired focus point; this attention is not directly associated with eye movements. These two types of attention allow another categorization of BCI spellers: gaze dependency (gaze-independent versus gaze-dependent). Many studies are seeking to achieve a gaze-independent speller that requires minimum ocular muscles movement. However, gaze-dependent spellers are most common.

Another characteristic of a BCI speller is the type of stimulus presented to the user. Most SSVEP systems rely on flickering stimuli with constant frequencies (each stimulus has its own unique frequency). P300 spellers, as mentioned before, are based on the oddball paradigm according to which characters are flashed periodically in a predefined order (pseudorandom). A number of them apply the oddball differently by animations, flashing faces, or movement.

Table 1 shows the taxonomy for the different BCI paradigms according to this categorization for the studies listed in this review. The filtered data resulted in 69 papers which meet all the filtration criteria. Of these papers, 45 are based on P300, 16 on SSVEP, and 4 each are based on MI and hybrid BCI. At the top of Table 1, the contribution percentages, from the total number of PRISMA results, of each BCI type are presented. The figure also categories the 69 systems according to the above-described taxonomy. This table could assist our readers to select the speller which falls into the category of interest or even find the appropriate speller on the basis of other characteristics. It also highlights some development opportunities, for example, none of the 45 P300-based spellers is asynchronous. It also underlines the features of each speller described in this review.

## 4. Review of BCI Spellers

Many types of BCI spellers have been developed over the years. This review primarily discusses the work done since the beginning of this decade considering the development of novel Graphical User Interfaces (GUI) of BCI spellers or improvements on the already existing and widely known GUIs.

As presented in the previous Section 3, over 400 publications were issued since 2010, with the aim of developing BCI-spellers. The PRISMA guidelines analysis showed that only ~18% of these studies directly targeted the improvement of the GUI design. Although other developmental aspects of a BCI system are very important to achieve a high-performing BCI-speller, the GUI is the first thing the end-user would encounter when dealing with such systems and it very often gets the least attention in the development process. In our opinion, the user-friendliness and the performance of the system are important factors. In addition, the design of the GUI might directly affect the performance parameters (accuracy and ITR).

In total, 75 relevant papers are discussed in this section of the review. The section classifies the spellers according to the type of BCI system used. This classification was presented for two main reasons: (1) Different types of BCIs might perform differently for the same user. The end-user might be interested in reading about a specific type of BCI, if from a previous experience he/she knows that this is the most suitable for him/her.; (2) Usually, each research team is working on a specific type of BCI paradigm. Categorizing these papers in this manner would also be beneficial for the readers. 

### 4.1. P300 Spellers Based on the Matrix Speller

**Figure 3:**
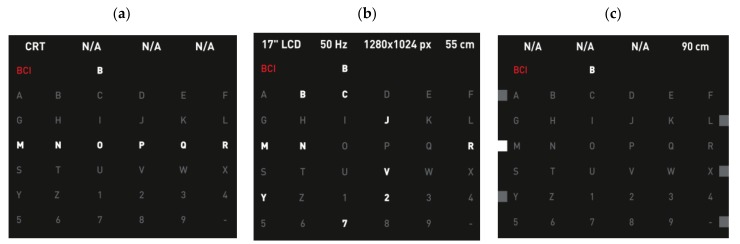
Graphical User Interface (GUI) of a modern P300 speller: (**a**) Matrix Speller inspired by the matrix developed by Farwell and Donchin in 1988 [7], shown during the intensification of the third row. (**b**) The random intensification similar to the one discussed in Yeom et al., 2014 [43]. (**c**) A view of the Edge Paradigm from Obeidat et al., 2015 [44] showing the intensification of the edge point next to the third row. All the above figures show “BCI” as the target word during spelling and “B” as an already selected character. Figures modified from the cited sources.

The first P300-based speller was introduced by Farwell and Donchin [7], and Figure 3a is showing a similar design to their GUI. It was the first BCI application based on P300. It consisted of a 6 × 6 matrix of flashing symbols displayed on a monitor. The items were organized in rows and columns (row–column paradigm, RCP), which were intensified in a random order, constituting an “oddball” paradigm. As this matrix consisted of six rows and six columns, at least 12 flashes were needed to flash each column and row once. The subject focused his/her attention on the target letter and was asked to count the number of flashes to help focus. The flashing of the row and the column which contained the desired target would produce a P300 wave in the EEG signals. The EEG signal was then processed, and the P300 signal was correlated to the order of occurrence of the flashing of the presented rows and columns. The analysis of these data resulted in the exact row and column which induced the P300 signal, the intersection of which was the selected letter.

The maximum accuracy reached in this study was 95% at a speed of 12 bits/min. This means a character can be selected from the matrix in approx. 26 s. This can be considered as very slow compared to conventional typing systems for healthy people; however, it can mean a lot for a person with no other means of communication.

The Matrix Speller is the base of most P300 BCIs. Researchers conducted many developments to make it faster, to achieve better classification, accuracy, and user-friendliness. The first research conducted by Farwell and Donchin had only four healthy subjects; however, over the years, many subjects (healthy and with different disabilities) have been testing their concept.

Farwell and Donchin proved the concept that P300 can be used for selecting a specific choice using the special arrangements of characters in the matrix, confirming that the P300 can be used for a communication application.

#### 4.1.1. Stimuli Variations

Many variations were proposed based on the GUI of the P300 Matrix Speller. One of the main variations is the change of the flashing stimuli. In 2010, Liu et al. [45] tested and discussed different types of intensification techniques for the Matrix Speller. Instead of just flashing individual symbols or rows and columns, as the flashing can be uncomfortable for some subjects, they used graphical effects like translations, rotations, zoom in/out, pattern rotation, and sharpening types. This stimulation technique can be applied to bigger menus with the advantage of a lower number of flashes, for a faster system. The different stimulation techniques suggested were a relative success. As a result, the best intensification was not the same for all subjects. This means the speller can be personalized individually for the best performance of each subject. Some types showed better results than typical flashing or a simple color change.

In [46], the 6 × 6 matrix speller was divided into four 3 × 3 submatrices. Randomly, the character was flashed from each submatrix once, so that, in total, only nine trials were produced. Another form of a submatrix stimulation was discussed by Eom et al. [47], called Sub-Block paradigm. Only a 2 × 3 submatrix of the 6 × 6 matrix speller was highlighted and not the entire row/column sequence. Further research showed that the change of the flashing patterns for individual characters was possible. 

In [48], only 7 or 9 flashes per trial were required, compared to the original matrix speller which required 12 flashes (one for each row and column), making the application faster. The nine flashes showed the highest accuracy and corresponding ITR, that were 92.9% and 14.8 bits/min, respectively, while the 12 flashes showed 88.0% accuracy and 10.1 bits/min ITR and the seven flashes showed 68.8% accuracy and 5.3 bits/min for ITR. The highest ITR reached was 17.3 bits/min, but with slightly lower accuracy. The aim was to achieve faster spelling speed and minimize the errors. A similar approach was shown by Polprasert et al. [49]. Similarly, the Random Set Presentation (RSP) was studied and tested by Yeom et al. in [43] to show the effect of a random intensification of characters, by flashing the characters in a random order (Figure 3b). 

Fazel-Rezai in [99] discussed the “adjacency problem”. Flashes next to the target seemed to be distracting the user and sometimes resulted in the wrong feedback as well as in the increasing of the problem of crowding, which refers to the difficulties in identifying a target if many similar objects surround it. In [43,46,47], the main aim was to avoid the adjacency-distraction effect and double-flashing errors. The system mentioned in [46] showed a higher performance than [47] with a mean accuracy of 99.70% and ITR of 26.8 bits/min. Dividing the 6 × 6 matrix into smaller matrices can be more comfortable for the users’ eyes, especially when only one character per submatrix was flashed at a time as discussed in [46]. In addition, in [43], the adjacency-distraction error was avoided by random-set representation, and, when flashing single characters randomly, no two adjacent letters were intensified at the same time.

The edges paradigm (EP) was introduced by Obeidat et al. [44] to overcome the mentioned challenges. The difference between the EP and the RCP presented in the Matrix Speller were the flickering points, which were added to the left of each odd row, to the right of the even rows, below the odd columns, and at the top of the even ones; the first step (row selection) is shown in Figure 3c. These points were intensified by increasing the illumination rather than by normal flashing, and the characters were fixed. For the selection of the desired letter, the subject first needed to focus on the edge of the row which contained the target letter. Then, during the second stage, the subject needed to focus on the edge point corresponding to the column which contained the target.

The edges paradigm was one of the most successful paradigms for solving the adjacency problem. As only the edges of the rows and columns were flashing, and not the characters, the flashing of characters was avoided, thus solving the adjacency problem and the double-flashing problem and reducing the discomfort which might result from an extended use of a flashing RCP. Although the mean ITR of the system was not as high as that of other presented systems, it still showed high accuracy. A total of 14 participants answered a questionnaire rating the levels of fatigue and comfort comparing the RCP and the EP. The results reported that the EP caused less fatigue and was more comfortable to use than the RCP. The advantages of this system were notable, and, as for the relatively low ITR, it can be adjusted by training, for example.

#### 4.1.2. Familiar Faces and Symbols

Numerous studies in the field of human face processing have revealed that the visual perception of familiar faces strongly involves several ERPs, which may be exploited for improving the classification. In particular, using faces well known to everybody in a given culture should lead to high and relatively stable effects across individuals. In [50], a 6 × 6 Matrix Speller was described; however, each character was superimposed by a semitransparent picture of a familiar (famous) face. In this study, they used faces of Albert Einstein or Ernesto ‘Che’ Guevara. The characters were intensified by the appearance of the familiar face behind the stimulated row or column. The paradigm was compared with a classic Matrix Speller where the new familiar face paradigm showed faster target selection and comparably high accuracy due to the fact that the familiar faces induced a higher ERP response.

The prototype in [51] was further studied in [52] by modifying the familiar face color to green. The green colored faces showed even a higher ERP response. A similar spelling system was used in [53] as a two-stimuli spelling system, utilizing familiar face and character flashing to increase the speed of spelling. The speller proved to be two times faster than the classical Matrix Speller. In [54], a similar approach was studied. A classical row/column paradigm and a random stimulus presentation of the row/column paradigm were compared to two proposed paradigms. The first paradigm presented random flashing of a self-face picture, while the second paradigm presented random flashing of non-self-face pictures. Another similar study was done more recently in [55].

Almost all of the mentioned spellers which use familiar faces showed a relatively high performance, higher accuracy, and faster ITR. The highest average ITR in this topic was ~80 bits/min reported in [53] with an accuracy of 81.25%. The highest mean ITR was reported in [54] with a 90.7% accuracy (more details, also about the other studies, are shown in the summary tables in the Discussion Section). The goal of using familiar faces was to have more effective visual stimuli which would elicit a stronger ERP signal. This would result in a more accurate classification. Also, combining the familiar face stimulus with another type of stimulus, like random-set-representation, was a promising development. It combined both systems’ advantages, avoiding main problems like adjacency-distraction and double-flashing errors.

In [56] a study by Kathner et al., rows and columns in a 5 × 5 matrix were flashed with the display of a yellow smiley face in a Virtual Reality (VR) environment. Using VR headset, two screens were tested: a full-view screen where the user could see the whole matrix and a second screen where the user could only see the part of the matrix on which he/she was focusing on, and head movement was required to visualize the rest of the matrix. It was tested on a patient with LIS, who showed adequate control over the BCI system. The paradigm combined with the Virtual Reality resulted in a fast and accurate BCI speller system. The system addressed one of the most challenging problems in the BCI field, i.e., portability. Using a virtual reality headset as a display eliminated the use of big computer monitors or other screens. The relatively decent performance was reported (see the summary tables in the Discussion Section). This BCI speller was based on a stimulus different from flashing characters; yellow smiley faces appeared over the characters for intensification. The mentioned stimulus type was similar to the familiar faces stimulation, resulting in a stronger ERP signal. The system performance was tested against the performance of the same interface on a 22” monitor, showing no significant differences. However, the second view proposed in the system, where the user had to move his/her head to see the rest of the matrix, can be impractical for some neuromuscular disease patients. 

#### 4.1.3. Variation of Letters Arrangement

Letters arrangement was tested as another parameter of the matrix speller. In [57], the arrangements of letters were changed according to the feedback from a built-in dictionary, which arranged the letters according to their usage frequencies. The more the user used a letter, the more accessible was the position in which it was placed.

In [58], the letters were arranged in a 7 × 7 matrix according to the frequency of their usage in the English language. Interestingly, for this system, the speller was tested with ten neuromuscular disease patients, plus ten healthy subjects. It showed higher accuracy than a normally ordered ABC interface, assuming less workload, but a lower ITR. From the questionnaires, most participants preferred the proposed interface to the ABC. The patients’ results appeared to have higher accuracy in the tested interface than in the ABC interface. The healthy participants had higher ITR in the ABC interface; however, ITR was not significantly different for afflicted subjects in the ABC interface and frequency-based interface.

Additionally, in Jin et al. [59], a laptop-keyboard-like matrix was suggested, where a 7 × 12 matrix was presented, in which the letters were arranged according to alphabetical order. The performance was remarkable (ITR 27.1 bits/min, accuracy 94.8% for 21 flash patterns); however, it was only tested on healthy subjects and it comprises many elements which might confuse some users who are not familiar with keyboards.

Inter-character distances were discussed by Sakai and Yagi [60]. The inter-character spaces in a matrix speller were changed and tested from 10 mm, to 25 mm, and to 40 mm. The results showed that the smaller the inter-character spaces were, the higher the P300 signal was. However, smaller inter-character spaces resulted in lower performance, as it was harder to classify the specific letter which caused the P300 response.

#### 4.1.4. Matrix Speller with Predictions

Another investigated approach to make the Matrix Speller faster and more efficient was the addition of a module to the speller which predicted and displayed suggested words for the user to select (for more information about integrating language models into BCIs, please refer to [61]). In 2011, Ryan et al. [62] developed an 8 × 9 matrix which displayed characters, numbers, and other commands on the screen, with the ability to predict the desired word and print suggestions from which the user could choose. A year after, Kaufmann et al. [63] provided a 6 × 6 matrix with predicted words and presented them as an extension of the matrix. The suggested words flashed within the matrix once they were predicted. The user could select the desired word in the same way a target letter would be selected. Later on, a modified matrix speller was added by Akram et al. [64,65], which included a built-in dictionary that displayed suggested words on the side after the user selected a few characters. Each word had a corresponding number. The second step of selection was via a 3 × 3 number matrix, where each number corresponded to one of the suggested words.

The study in [62] was aiming for high ITR without affecting accuracy by adding prediction words. However, the authors suspected that adding a predictive module to the speller might be more cognitively demanding for users because of multi-tasking. This study showed lower accuracy than the usual matrix speller paradigm, but higher ITR. It was indirectly deduced that a predictive system increases the workload on users which can decrease the P300 signal amplitude. This can be enhanced by the training of both, the users and the predictive dictionary. In [63], the solution to the workload problem accompanied with a predictive speller was proposed by displaying the suggested words as part of the matrix. This resulted in less workload on the BCI users, was more comfortable, and also showed a better performance. The system can be modified further to recognize grammar rules and fill in some words for the user. Overall, the spellers with predictions discussed in this review showed promising results, higher ITR than most of the matrix spellers, and a reasonable accuracy (further details in the summary tables in the Discussion Section).

#### 4.1.5. Other Languages

Other P300 speller developments were made by including more interfaces for different languages to allow a broader group of people to benefit from such BCI applications. Developing innovative interfaces for languages based on script was also really important, as it might take a long time to type one word using a BCI speller. Also, most of these non-English interfaces proved to achieve a performance comparable to that of other English spellers.

• Chinese

The Chinese language has a logographic script comprising more than 11,000 characters which are based on strokes. A P300-based BCI has been developed that allows users to input Chinese characters stroke-by-stroke [100]. However, this was not very efficient, as a single Chinese character may consist of 20 or more strokes, and took a long time. Minett et al. [66] showed how a P300 matrix could present an efficient way to type Chinese characters. Also, refs. [67,68] have developed Chinese BCI spellers. 

• Arabic

In Kabbara et al. [69], a P300 Matrix Speller was presented in Arabic letters for the first time. A 6 × 5 matrix displays all the Arabic letters in an RCP, where the intensification was the random flashing of rows and columns.

• Korean

In 2011, the first Hangul (Korean script) P300 speller was developed [70]. Hangul has a hierarchical structure entirely different from English; therefore, a two-stage speller was needed. Two-stage speller means that there are two different screens displayed, dividing the symbols.

• Japanese

A conventional Japanese P300-based BCI spelling system consisted of a 6 × 10 matrix. However, Yamamoto et al. [71] proposed a two-phase matrix system; each phase is a 6 × 5 matrix with the option to move between them. This solved the crowding problem as well as decreased the number of flashes needed per trial. A similar approach, another two-phase speller, was tested on ALS patients in [72] and compared to the performance of the conventional system. The two-phase speller was successful in producing higher accuracy rates when tested with ALS patients.

#### 4.1.6. 3D Blocked Matrix Speller 

In Noorzadeh et al., 2014 [73] a 3D virtual matrix was suggested. The characters were displayed in 3D blocks instead of the usual 2D screen arrangement. Different flashing techniques were tested for this new design. The study confirmed that this arrangement has the potential to be a more user-friendly GUI.

The speed was proven to be higher in the proposed 3D interface compared to the classic 2D interfaces, since it needed a smaller number of flashes. However, 3D interfaces might need higher computational power. However, still, a 3D design is more attractive and user-friendly.

### 4.2. Other P300 Interfaces

In this section, other P300 interfaces, which were not a direct development of the original matrix speller, are described and discussed.

#### 4.2.1. Chroma Speller

**Figure 4:**
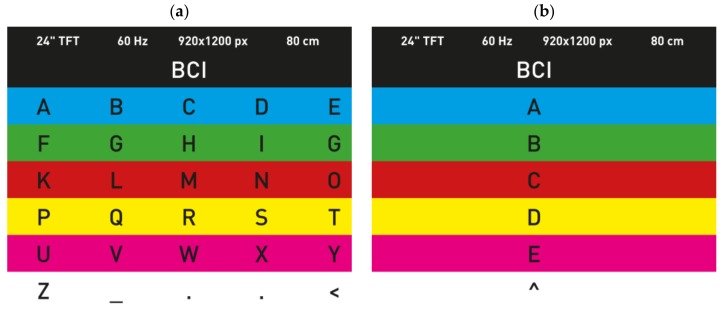
Chroma Speller in its two operating stages [74]. (**a**) The first-stage selection. (**b**) The second stage to select an individual character. The target word here was suggested to be “BCI”, and “B” is the target letter. Figures modified from [74].

The Chroma Speller, developed by Acqualagna et al. [74], worked via presenting six differently colored stimuli on a black background, as shown in Figure 4a. A total of 30 characters and symbols were grouped into the six colors for the first selection. When it started operating, the colors flickered in a series manner. The subjects had to focus on the desired color to select it, and the ERP P300 signal was detected and analyzed. After the first selection of a group of characters, the individual characters of the selected group were presented separately on the second screen with row colors similar to the first display, as shown in Figure 4b, with the option to go back to the primary group display if the white box was selected.

The Chroma Speller aimed to achieve a gaze-independent speller system with a minimum workload, as the user had only to focus on the required color (the color which contained the desired character) and not on the individual letter. The proposed system was compared to the Centre Speller (refer to Section 4.2.7.) during the study, showing a higher performance. Consequently, this speller was undoubtedly suitable for patients in advanced stages of ALS, as they face a limited oculomotor control. In addition, the system included an auditory feedback citing the selected letter, which can be helpful for these patients as well. However, on the basis of our knowledge, such system has not been tested yet by ALS patients.

#### 4.2.2. T9

**Figure 5:**
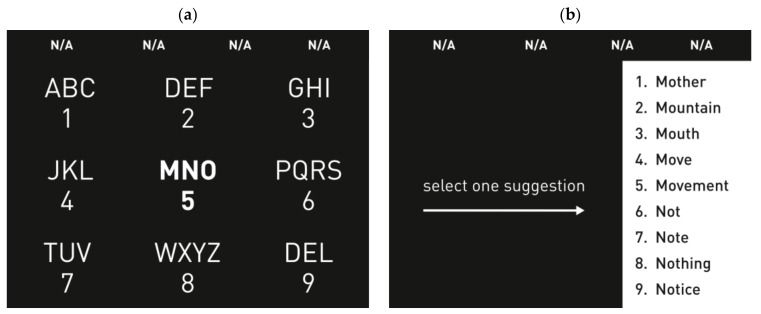
The GUI discussed in [75]; (**a**) The first stage where a target letter was selected. (**b**) Suggested words were displayed with the corresponding number. Figures modified from [75].

The first time a T9 (text on nine keys) paradigm was presented as a BCI speller, it was based on auditory stimuli [101]. A modified, visual-based stimuli T9 speller system was introduced in 2015 [75] and is shown in Figure 5, with an integrated dictionary to propose suggested words to save time. T9 is the same approach used in early mobile phones for texting on the number keypad. In this study, they used only eight keys for character input and one as a delete option in case of errors. The user started by typing a few characters of the desired word, then selected one of the suggested words from the same 3 × 3 matrix in the GUI. The targets were highlighted randomly. Figure 5a illustrates how the selection of the letters group occurred, and Figure 5b the second stage, where the selection of a number for the first screen corresponded to one of the suggested words in the list on the right-hand side. This paradigm reduced the typing time significantly, especially compared to other multi-stage spellers. The fast typing speed was credited to the word prediction module embedded in the interface, which was not present in the traditional Matrix speller. Also, as a result of a lower number of stimuli in a T9 interface, the speller was more user-friendly and might have caused less fatigue. As another advantage of having only nine targets, the eye movement was minimized. 

A similar T9 system was used in another study in [76] to test its performance with ALS patients and to compare it with a modified Matrix Speller. The T9 showed a faster typing rate with ALS patient compared to the Matrix Speller, revealing a promising performance.

#### 4.2.3. Checkerboard Paradigm 

**Figure 6:**
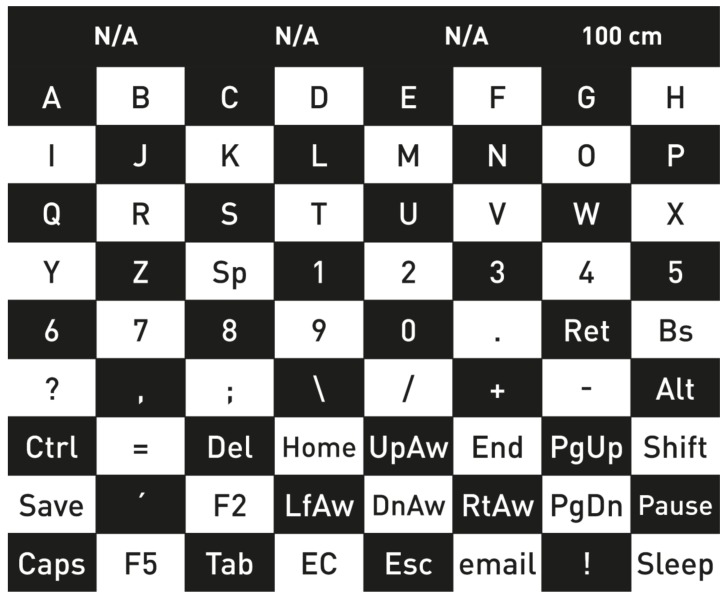
Checkerboard paradigm similar to the one studied in Townsend et al. [102]. Figure modified from [102].

The “Checkerboard” paradigm (CBP) was proposed in [102]. As presented in Figure 6, it was composed of a 9 × 8 matrix of characters and commands, and, to avoid the “adjacency-distraction problem” and the “double flash” issues, the sets of nonadjacent elements were pseudo-randomly flashed [102]. Also, the same paradigm conducted on ALS patients showed higher online accuracy rates for the CBP. 

The half checkerboard paradigm (HCBP) [77] divided the matrix on the screen into two separate regions: “left” and “right”. The authors also used electrooculography (EOG) to identify the eye position, so that only the characters in the eye-gaze area would start flashing. This paradigm was targeted to people who can voluntarily gaze at a target and to disabled people who still retain some eye movement. When an area was selected by gazing, it flashed half of the presented 72 targets. The performance of the HCBP was compared with that of the Checkerboard paradigm, resulting in higher accuracy and faster information transfer rates.

The aim of the Checkerboard Paradigm [102] was to compare a new representation of a P300 speller with the RCP. Although the performance of both systems was almost the same, the CBP proved to be less affected by the common errors faced when dealing with RCP. Also, the system was supported by successful trials with ALS patients with better results than with the RCP. Moreover, the participants shared their opinion about the CBP, stating that it was more comfortable and caused less fatigue.

#### 4.2.4. Geospell

**Figure 7:**
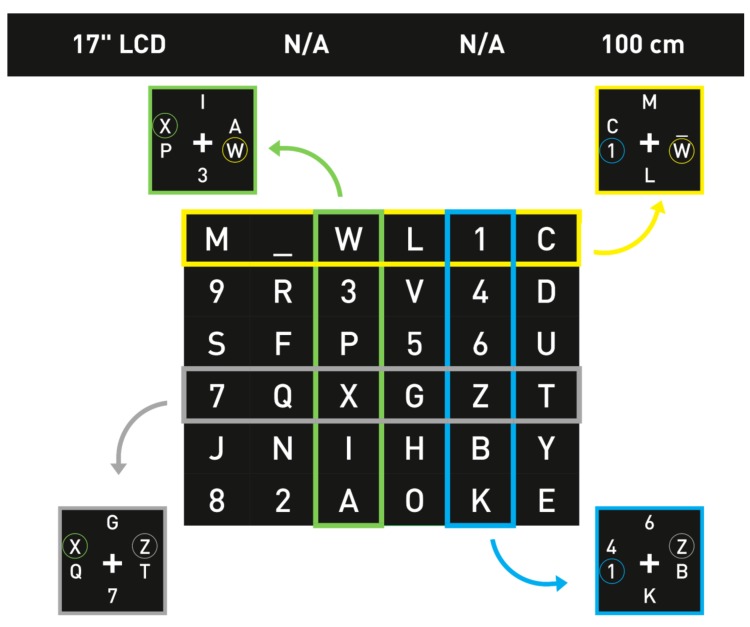
The GeoSpell as discussed in Aloise et al. [78], showing the group organization concept. The figure is modified from the original source [78].

We can consider the GeoSpell (Geometrical Speller) as a rearrangement of the P300 Matrix Speller. It was developed by Aloise et al. [78], focusing on covert attention speller. The main concept was to use an N × N matrix, for example, a 6 × 6 matrix, where the total number of characters is N^2^ (6^2^ = 36 characters). Then, the matrix layout was transformed into 2 × N sets of square frames, each containing N characters. In addition, the rows and columns were re-arranged, so that each was displayed in a separate box; Figure 7 shows the arrangement. Therefore, each character existed in two sets: one corresponding to the row, and the other corresponding to the column. Each set appeared and flashed on the screen at a fixed point in the center to help the subject to avoid eye movement (an eye tracker was used to track gaze positioning). The identification of the target character was based on the classification of the two sets in which the target character appeared. However, the system did not show great performance to compete with typical BCI spellers. A similar approach was already discussed before in [79]; however, in this study, two different arrangements for the sets were tested (without an eye tracker). 

This interface was declared to be a gaze-independent transformation of the matrix speller. Even though the accuracy was similar to the RCP, the typing speed was low compared to other spellers. This was mainly due to the number of different frames which were displayed in each trial. 

The same team who presented the original Geospell system carried on further research on the same speller a couple of years later to investigate the unexpected low performance obtained in 2012 [103]. They compared the performance of the Geospell during covert attention with that of the Matrix Speller with overt attention. The aim of the study was to find an explanation, i.e., why the performance during covert attention was lower than during overt attention. The authors concluded that the overt attention modality was more accurate than the covert attention one, as it was cognitively more demanding. However, they also mentioned that, with some compensations, the Geospell could be equally or more accurate than the Matrix speller. 

Modifications of the GeoSpell were developed and discussed in [82]: Motion-Covert GeoSpell (MCGS) and Covert GeoSpell (CGS). The purpose of this study was to investigate the performance of mVEPs for multi-objective gaze-independent BCIs. MCGS used motion-flash stimuli where characters appeared and moved a fixed distance to the edge of the screen during the presentation, while CGS only used the usual flash stimuli. Both systems were tested under the covert attention condition. The offline results showed that a higher P300 was evoked by the CGS compared to MCGS. This study concluded that mVEP could not enhance the performance of multi-objective gaze-independent BCIs regarding ERP. 

#### 4.2.5. Gaze-Independent Block Speller (GIBS)

**Figure 8:**
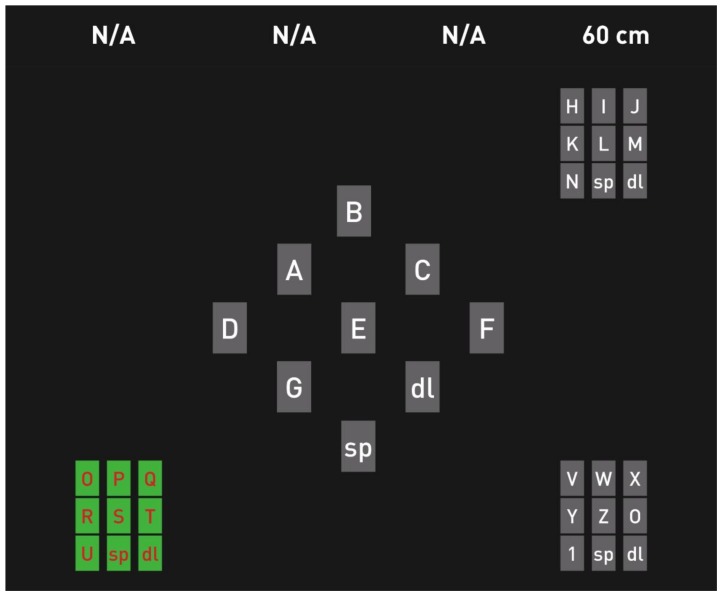
GIBS as discussed in [80]. Figure modified from [80].

The paradigm in [80] targeted the problems of covert attention and it was based on P300 BCI. The presented GUI had 30 characters, as shown in Figure 8. The symbols were grouped into four blocks which were located at the corners of the screen. Group 1 = [A B C D E F G], Group 2 = [H I J K L M N], Group 3 = [O P Q R S T U], Group 4 = [V W X Y Z 0 1]. The stimulation consisted in the flashing of the different blocks. The selected block was then expanded in the middle of the screen in a diamond shape (Figure 8). The second stage was presented by the flickering of the individual characters in the shape of a diamond. When the symbols were expanded to the center, they were larger and far apart to avoid crowding. The results showed that GIBS can be used without ocular movement. Moreover, by using bit rate analysis, the authors showed that GIBS could produce similar information transfer rates when compared to the standard Row-Column (RC) speller. 

GIBS was another gaze-independent P300-based speller, which also tried to avoid the matrix layout that has been proved to face some challenges. Being a two-phase system, fewer targets flickered per display. 

#### 4.2.6. Lateral Single Character Speller (LSC) 

**Figure 9:**
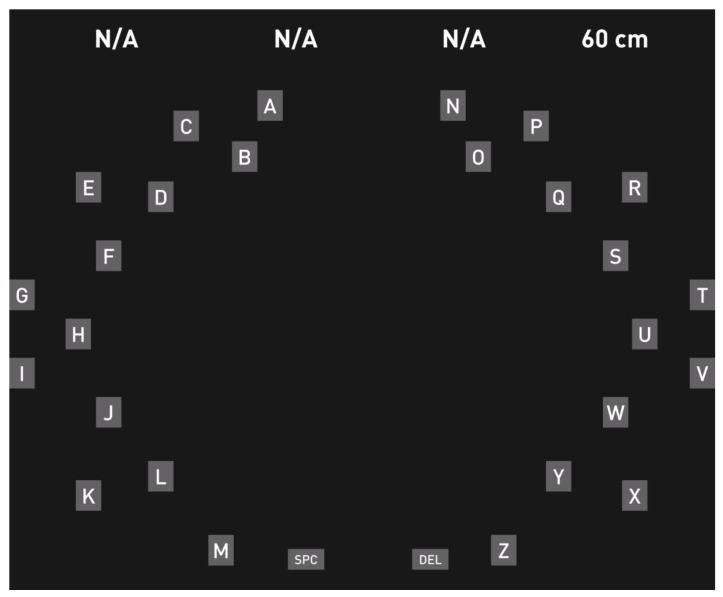
Lateral Single Character Speller, similar to [81]. Figure modified from [81].

Pires et al. [81] proposed a lateral single character speller that was compared to other RC spellers; the layout reduced the effect of the local and remote distractors. Furthermore, the paradigm was expected to be more visually attractive and comfortable. The proposed Lateral Single Character Speller (LSC) speller, shown in Figure 9, contained the 26 letters of the alphabet and the ‘spc’ and ‘del’ commands. The 28 symbols flashed alternately and pseudo-randomly between the left and right fields in a lateral and symmetrical arrangement. The user had to focus only on one side of the screen, looking at one half of the display at a time. The target word for the copy task and the selected letters were shown in the middle of the arrangement, which required a short eye movement by the user. 

As the interface divided the targets into two separated groups, it avoided the crowdedness problem as well as the adjacency-disturbance error, which occurred in the RCP because of the flashing of many letters, which were located close to each other. The paradigm showed better performance than the standard RC speller and it was effective with ALS patients and other patients with neuromuscular diseases (ITR 26.11 bits/min and accuracy 89.90%). From the questionnaires, the test subjects reported a preference towards the LSC, stating that it was more comfortable.

#### 4.2.7. Hex-O-Spell as ERP

The Hex-O-Spell, a gaze-independent BCI speller that relies on imaginary movement, was first developed in 2006 by Blankertz et al. [39] and also presented in [104] (described in details in Section 4.4.1). This type of BCI has inspired many researchers to develop new BCI spellers. Here, we discuss some variations of the original Hex-O-Spell [39]. These variations were mainly developed to study the possibility of gaze-independent BCI speller systems which can be useful for late-stage ALS patients. 

**Figure 10:**
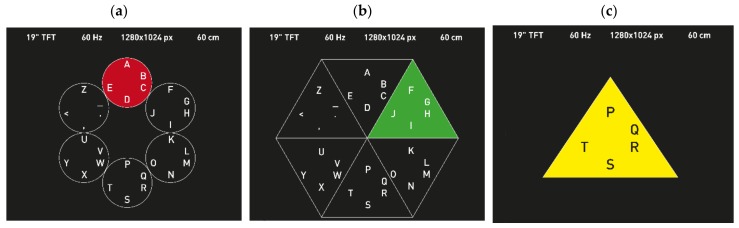
Three variations of the Hex-O-Spell with ERP for gaze-independent BCI studies. (**a**) Hex-O-Spell; (**b**) Cake Speller; (**c**) Center Speller. Figure modified from Treder et al., 2011 [83].

The first variation of the Hex-O-Spell was mentioned in [105], to be used as an ERP P300 BCI system, to test if there was a difference between the system’s performance during covert attention and overt attention. In this study, a Hex-O-Spell, with minor changes with respect to its GUI, was compared to an adapted Matrix Speller. The modified Hex-O-Spell had circles around a central invisible hexagon instead of hexagons around a circle (similar to Figure 10a). The intensification was done with size changes. The size of the characters in the circle and the circle itself increased in turn, one by one. The Hex-O-Spell showed higher accuracy and a higher ERP response than the Matrix Speller in both covert and overt attention conditions. 

Other variations of the Hex-O-Spell utilizing ERP systems are the Cake Speller and Center Speller [83]. These two GUIs were developed to be compared with the Hex-O-Spell ERP in [105] for gaze-independent BCI spellers. During this study, a different intensification technique was used for the Hex-O-Spell ERP (Figure 10a). Instead of only changing the character size for intensification, the fill-in color of the circles was also changed, from the standard black background to one of six different colors (each circle had its own color). The Cake Speller was composed of a central hexagon, which was divided into six equal triangles, as shown in Figure 10b. Each triangle contained five characters, and the triangles were intensified one by one, by changing the fill-in color, using the different colors assigned to each triangle. Once one of the triangles was selected, the characters expanded and were distributed to the six triangles for the second stage of the selection.

The Center Speller is shown in Figure 10c. In this BCI speller, each group of characters (also five per group) was represented alone in the center of the screen, within a colored geometric shape. The combinations of letters and symbols within the geometric shape were called “elements”. Each element was presented individually in the middle of the monitor with a unique color and inside a unique geometric shape. After the first stage of selection, the individual letters filled into the groups, in each element for the second-stage selection. To clarify, for each selection stage, six different displays were presented. The character intensification, in this case, occurred simply by the appearance of the element in the screen. A later study combined the Center Speller with the Hex-O-Spell, to create a test- and error-detection approach [84]. This paradigm can also be considered as an RSVP, which is discussed in the next section. 

In [105], the Hex-O-Spell was transformed into an ERP system, to test whether ERP spellers could also be gaze-independent or not, with the aim to check if BCI-spellers could substitute eye tracker speller systems. Although the accuracy with covert attention was relatively low, it still proved that the ERP speller could operate with covert attention only and that accuracy could be improved in further studies. However, the Hex-O-Spell showed a higher performance during covert attention than the Matrix Speller. This concluded that the change in the design could improve the performance of the speller and grant a more effective control without the need for spatial attention. However, it still has to be tested on ALS patients. Other variations mentioned in [83] were also developed with the primary purpose of achieving gaze-independent spellers, such as the flashing in different colors as an intensification technique to elicit a stronger ERP signal. Although the typing rate was relatively slow, all the suggested interfaces showed high accuracy compared to other spellers (especially gaze-independent spellers). This could be treated as a proof of concept that ERP spellers can be effective without the need for gaze attention.

#### 4.2.8. Rapid Serial Visual Presentation (RSVP)

RSVP was developed with the aim to form an efficient gaze-independent ERP speller. The paradigm was quite simple: individual characters appeared in the center of the screen in a randomized manner. The target letter evoked an ERP signal when it appeared. It was first presented in 2010 by Acqualagna et al. [85]. In this study, two variations were presented, a monochrome one and a colored one. A total of 30 characters (letters and punctuations) were presented. In the colored version, the characters were divided into three different colored groups: red [A B C D F G H I J −], green [K M N W E Q R S T +], blue [U V O X Y Z L P ! /]. To prevent symbols from clustering together frequently, pseudo-randomization was applied on the order of presentation, and, to allow for significant behavioral data to be obtained, the number of occurrences of the target symbol varied before and after each trial. The user had to look at the screen and count the number of times the target letter appeared in the middle of the display. The subjects showed better performance with the colored letters than with the monochrome ones. The accuracy of the RSVP speller outperformed both that of the Matrix speller and that of the Hex-O-Spell. 

In 2011, Acqualagna and Blankertz [86] investigated three variants of the RSVP paradigm GUI, with different colors and different speeds of character representation. The performance of this paradigm was also tested online in 2013 [87].

Although the accuracy of the RSVP was very promising in all mentioned studies (around 94% on average), the ITR was lower than expected. The reason was that the user had to wait for the target letter to show up in between the rest of the 30 characters. This waiting wasted time and might have caused the user to feel bored or to lose focus. However, the system worked as a gaze-independent speller.

In [88], a new visual ERP-speller using N100 in addition to P300 was proposed. N100 is a type of visual evoked potential (VEP) which is induced by paying attention to the visual stimulus, and is not related to the oddball paradigm, making it difficult to use N100 alone for BCI. The authors in [88] claimed that this was the first time where N100 was used for BCI commands classification. In the proposed system, P300 and N100 were used separately and independently to determine the target character and to overcome the familiar challenges of an ERP speller. The GUI was similar to the standard 6 × 6 P300 speller with 36 commands: 26 letters (A–Z) and 10 numbers (0–9). However, the stimulus presentation was based on rapid visual presentation (RVP) to enable the implementation of the N100 into the BCI. Two BCI systems were developed, 2 × 2 and 2 × 3 matrices, which were presented as an RSVP stimulus. Each layout contained a group of the characters arranged in fixed positions. The assumption was that the user knew beforehand the position of the target letter. In the case of the 2 × 2 matrix, one position was left blank for three of the 12 different stimulation images in order to elicit the N100 signal. For the 2 × 3 matrix, nine stimulus images were used with similar blank positions. The input speed was faster than that of the P300 speller, as only nine stimulation sequences were required. The classification occurred by combining the P300 signal with the corresponding N100 from the blank position.

Eleven healthy 22–24-year-old males participated in this experiment, and performance comparisons between the two presented layouts and the P300 speller were carried out. For the 2 × 2, the average accuracy for the P300 was 63.1%, while the proposed speller in [88] showed an average accuracy of 74.7%. The average ITRs were 0.53 bpm and 0.70 bpm for the P300 and the proposed system, respectively. As for the 2 × 3 layout, the accuracies were 67.8% and 70.3%, and the ITRs were 0.60 bpm and 0.85 bpm for the P300 and the proposed system, respectively. The introduction of the N100 provided a one-stage selection and, as a result, it reduced user’s fatigue and improved the accuracy of the system. However, it required the user to memorize the position of each letter beforehand, which might be difficult for some potential users. 

### 4.3. SSVEP Spellers

An advantage of the SSVEP approach is that it does not require calibration or subject training. In addition, SSVEP spellers should be generally faster than P300 spellers, as no specific number of trials are required for them. A target can be selected as long as the signal is strong and stable enough to be detected by the software. 

#### 4.3.1. Bremen Speller

**Figure 11:**
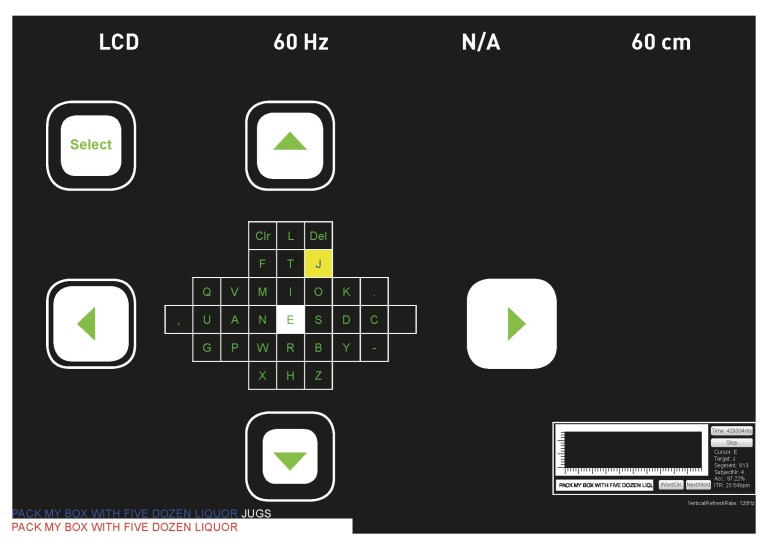
A similar GUI to the Bremen-BCI Speller during the selection of the right arrow, as the box size is increasing during selection [106]. Figure modified from [106].

**Figure 12:**
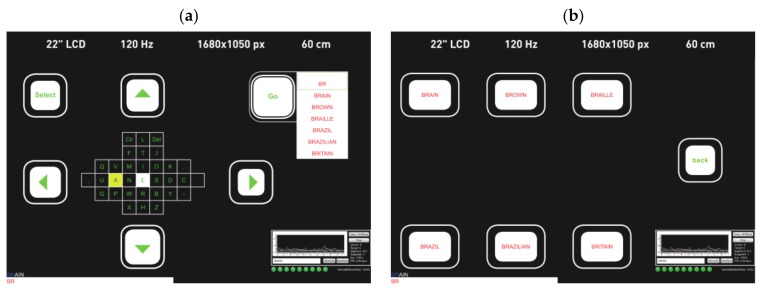
(**a**) The modification of the original Bremen-BCI speller when a build-in dictionary was added to it [28]; (**b**) The second stage of the GUI, where suggested words were presented to the user to choose the desired word.

One of the earliest high-speed SSVEP-based BCI spellers is the Bremen-BCI speller [106]; a similar GUI is shown in Figure 11. In this study, a virtual diamond-shaped keyboard containing 32 characters was presented. The five boxes (four with arrows and one with the command “Select”) were used to control the movement of a cursor which could move along the characters and select the desired target. Each of these boxes flickered with a certain frequency to elicit an SSVEP response. The letters were arranged according to their usage frequency in the English language. At the beginning of each trial and after each selection, the cursor was located by default in the middle, over the letter “E”. The system gave audio feedback to the user, i.e., the system announced the selected letter out loud, so that the user or anyone nearby could hear it as a selection confirmation. Later in [28], a built-in dictionary was added to predict the desired words, as well as another type of feedback to notify the user about the selection, consisting in the size of the white boxes varying according to the power of the SSVEP signal, e.g., when the SSVEP signal increased, the size of the box increased, to notify the user that a selection was about to be made. Figure 12 shows the addition of the prediction module. It consisted of two different stages (layouts). The first stage was similar to the previous Bremen-BCI Speller with an extra sixth box with the command “Go” (Figure 12a). After the selection of at least two letters, a drop-down list of six words suggested from a dictionary appeared next to the “Go” command. If one of these choices was the desired word, the user had an option to select “Go”. This action would lead to the next layout where each of the suggested words was presented in a flickering box (Figure 12b) and could be selected by the user to be written. 

The Bremen-BCI Speller had gathered over the years a remarkable number of subjects. In addition to hundreds of tested healthy subjects, 37 participants were recruited during the RehaCare rehabilitation fair, eight of them with different disabilities [106]. Each participant took part in five different spelling tasks. The average ITR reported was 25.67 bits/min, with an accuracy of 93.27%, which indicated a competitive performance, especially for patients with neural malfunctions. Of note is that the experiments were carried out during a rehabilitation fair with a high level of noise and surrounding distractions. As for the Bremen Speller with the built-in dictionary, it showed a faster performance when compared to the original speller, with 32.71 bits/min and 29.98 bits/min, respectively. As another advantage, the dictionary implemented kept track of the most commonly used words. This feature speeded up the spelling by proposing the most often used words first. Also, it is worth mentioning that this speller was the first SSVEP-based speller with the option to predict words. After further improvements in signal processing, an average ITR of 61.70 bits/min, with a peak of 109.02 bits/min, was achieved with the Bremen-BCI speller, in a test with seven participants [107].

#### 4.3.2. Multi-Phase SSVEP Spellers

We presented a three-phase SSVEP speller in [29]. This study aimed to investigate the performance’s differences of SSVEP-speller according to the subjects’ age. The GUI consisted of four flashing white boxes with green characters or commands inside them, on a black background. Only the white boxes flashed while the green text was fixed. One of the boxes showed the command “delete”. The other three boxes contained the letters of the alphabet. On the first screen, nine characters per box were displayed. When one box was selected, its content was spread over three boxes to form three characters per box. During the last selection phase, when a box was selected, the three boxes contained one letter each. In the second and the third stage, the “delete” box changed to “back” to give the option to go back to a previous layer in case of error. Every selection gave an audio feedback, naming the selected box. A similar approach was presented earlier in [30].

In [29], all subjects from two groups, a young age group and an older group, achieved control over the BCI system. The mean values of the young group were 98.49% accuracy with ITR of 27.36 bits/min, while the older group’s accuracy was 91.13% with ITR of 16.10 bits/min. Although there was a significant difference between the performances of these two groups, the system was reliable with relatively high performances.

In another study, Cao et al. [31] proposed an SSVEP-based speller system with two phases. This speller would allow the input of 42 characters comprising of letters, digits, and symbols. Its user interface had three pages and 16 targets on each. Page turning was done via two boxes (buttons) which previewed the characters on hold on their corresponding page, aiding the user. Another two-phase SSVEP speller was discussed in [98] and compared to the Bremen speller. In this study, we presented a GUI with five boxes; each contained six alphabet characters and special symbols. Two other boxes were present, one containing the commands “delete” during the first window and “back” in the second window, and the other with the command “Clear”, where the user could delete the whole word. When a box was selected, the content of this box was spread out to form one letter or symbol per box, in the second window. Another recent multi-stage SSVEP speller was presented in [32]. 

The two-phase SSVEP spellers may have a higher performance than the three-phase spellers, as fewer steps were needed for letter selection. In [98], comparing the two-stage SSVEP speller with the Bremen-BCI Speller, most subjects stated that the two-phase speller was more user-friendly than the Bremen Speller. However, the mean values for both spellers regarding ITR, accuracy, and time did not show any substantial difference for any of the tasks.

All the rest of the SSVEP mentioned studies proved remarkable performances, with the highest mean accuracy of 98.78% presented in [31] and mean ITR of 61.64 bits/min. However, none of the mentioned studies, except the one about the Bremen Speller, included MND patients as subjects for testing the system.

#### 4.3.3. Multi-Target One-Phase SSVEP Spellers

Multi-Phase SSVEP spelling systems typically utilize a low number of distinct stimuli. The number of stimuli is anti-correlated to the number of phases. A low number of stimuli results in a low spelling speed, as classification and gaze-shifting phases of each phase are accumulated. Several groups, therefore, developed spelling applications that employ multiple stimuli simultaneously. This allows letter selection in a single step, resulting in much higher spelling speeds.

Wang et al. proposed a method to realize multiple SSVEP stimuli on computer screens [33]. The method was initially tested online with a virtual keypad consisting of 16 SSVEP target stimuli. The three subjects achieved an ITR of 75.4 bits/min, with an average accuracy of 97.2%.

Meanwhile, the methods were further improved and led to the highest ITR values reported for BCI spellers. As a result of refined classification methods and user-specific calibration data, Chen et al. achieved average ITRs of 267 bits/min and accuracy of 89.76%, employing 40 SSVEP targets [34]. The stimuli were arranged as a 5 × 8 matrix containing characters, numbers, and additional symbols. 

Recently, Nakanishi et al. reported an average ITR of 325.33 bits/min in a cue-guided task using a 40-class speller, with an accuracy of 89.83% [89]. It was also stated that free spelling resulted in a slightly lower ITR (198.67 bits/min) and that inexperienced users required longer gaze shifts. In general, a higher number of targets in SSVEP-based BCI increases the spelling speed but also increases eye fatigue and target misclassification.

Multi-target BCI spellers have also been realized using the c-VEP paradigm. Spüler et al. achieved an average ITR of 143.95 bits/min with nine subjects [35]. The 32 target stimuli were arranged as a 4 x 8 matrix and were used to select letters, numbers, and underscore. Wei et al. tested a 48-target c-VEP system with four participants and achieved an ITR of 129 bits/min [36]. In c-VEP-based BCIs, all stimuli share the same circular shifted code pattern. Thus, the spelling accuracy requires precise timing between stimuli presentation and data acquisition. 

Recently, Nagel et al. investigated the effect of monitor raster latency [108]. By correcting the raster latency, the distance between the most probable and the second most probable target was increased by 18.23%, resulting in a more reliable system.

#### 4.3.4. RC SSVEP Speller

A dynamically optimized SSVEP brain–computer interface speller was presented in [90], which emulated the stimulation technique of a P300 Matrix Speller. A row/column (RC) paradigm was introduced into the SSVEP BCI to create an SSVEP speller with 36 items, flickering with only six frequencies, one for each element in a row or a column. A similar stimulation approach, which combines the P300 with SSVEP, was previously discussed in [91,92] as well. In [93], a SSVEP and P300 combination was also used; however, the P300 stimulus was different. The autors applied changes in the color, size, and rotation of the characters for stimulation.

#### 4.3.5. FlashType^TM^

The FlashType^TM^ [37] is one of the newest c-VEP BCI spellers. This type of speller does not rely on selecting individual letters like the previously discussed spellers. Instead, it controls the movement of a cursor and selected letters or symbols from a static keyboard. In the center of the displayed window, the keyboard had 28 visual targets; a row above showed the suggested character to be selected, and another row at the top showed the suggested words to be typed. The arrangement of the user interface was designed to utilize the majority of the screen and also to maximize the inter-stimuli distance. In the four corners of the screen, the stimuli were presented in four green/red 5 × 5 checkerboards with two patterns each. The shifting between the two patterns was according to a special pseudorandom binary code, which resulted in an induced Coded Visual Evoked Potential (c-VEP) signal. The four stimuli presented four different controls to the cursor: select, horizontal movement, vertical movement, and reverse. The horizontal and vertical controls pointed the movement of the cursor in the desired direction, while the select stimulus selected the target letter. The reverse stimulus moved the cursor in the opposite direction relative to the default direction in the horizontal or the vertical mode. The first character from the left on the Character Suggestions row was the cursor’s default starting point. Then, the subject proceeded by moving the cursor vertically to select the looked-for row. The active row was marked in a yellow frame. After row selection, horizontal movement was required to select the wanted column; an active column was marked in a purple frame. The selection of the column resulted in the selection of the target letter, which fell in the intersection of the selected row and column. 

Another mode of operation, which is still under further study, is the auto-scroll mode. This mode was developed to give minimum to no gaze-dependency. During auto scroll, only one stimulus was active, the select stimulus. The cursor moved automatically, stopping at each row and column in a specific order. All that the subject needed to do was to “select” the target while the cursor was pointing at it. Although that mode might be helpful for patients with no eye movement control, it is extremely slow.

This study reported notable advantages. The experiment was conducted using only one electrode to read the signal for the four stimuli which aided the system to be more user-friendly, as less preparation was required. It also classified the system as being relatively more portable compared to other BCIs which require eight or more electrodes. The performance results showed high accuracy and relatively fast typing for all three subjects. Moreover, a significant advantage of the static keyboard was that the characters could be substituted for characters of any language or even replaced by communication symbols. The predictive words option made the speller faster and more user-friendly. In addition, the speller did not require a lot of eye movements as the subject needed to only move attention to the four stimuli controlling the cursor and not to each character. Plus, the added mode of auto-scroll could be relatively slow, however, it would be beneficial for patients without oculomotor control. On the other hand, the interface was only tested by three healthy subjects, which is a small number compared to other studies, especially modern studies. In addition, none of the participants were NMD patients.

#### 4.3.6. DTU BCI Speller (Technical University of Denmark)

DTU BCI is an SSVEP-based BCI developed by Vilic et al. [38]. The typing area divided the screen. On the left-hand side, there were seven stimuli. Each of them corresponded to a group of symbols, which was active during the first stage of spelling. During the second stage, the stimuli boxes on the right-hand side started flickering. These boxes presented the suggested words from the built-in language model dictionary. Another flickering box was situated at the bottom of the typing area. This box was always active. It permitted the user to voluntarily choose to switch from the first spelling stage to the next. Once a target was selected, it turned green for few seconds to give a feedback to the user. When a word was chosen, space was added, and the stimulation was active on the left-hand side again, starting automatically. 

The users gave a positive feedback regarding the friendliness of the interface. As only three electrodes were used, the setup time was minimal, and the portability of the system was realistic. The added built-in dictionary for word prediction supported the users to reach faster typing speeds using this BCI speller. The average overall performance of this system was reasonable compared to other SSVEP spellers (ITR 21.94 bits/min and accuracy 90.81%).

### 4.4. MI Interfaces 

In this section, we present different spellers which are based on MI. A unique feature of MI-based systems is that they are not dependent on any kind of external stimuli.

#### 4.4.1. Hex-O-Spell

**Figure 13:**
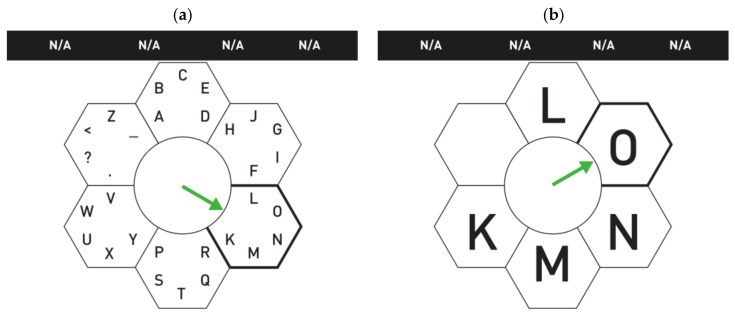
The GUI similar to Berlin Hex-O-Spell GUI, as shown and discussed in Blankertz et al., 2006 [39] (**a**) during the first stage of selection and (**b**) the second stage for selecting an individual letter. Figures modified from [104].

The Hex-O-Spell, a gaze-independent BCI speller that relies on imaginary movement, was first developed in 2006 by Blankertz et al. [39], also presented in [104]. It was inspired by a mobile device, which relies on the change of the device orientation for typing. The aim was to develop a working, synchronous BCI system, with the least number of controls possible (two) for 30 targets (26 letters + punctuations). The two controls were based on two mental states: imagined right-hand movement and imagined foot movement. As shown in Figure 13a, six hexagons were arranged eccentrically around a circle containing an arrow pointing out from the center towards the hexagons. The 30 characters were divided equally among the hexagons, five characters each. By imagining the right-hand movement or foot movement, the subject could rotate the arrow or select the hexagon that the arrow points to, which contains the target letter, respectively. In the second stage of selection (Figure 13b), the characters from the selected group spread out in a way that each letter or symbol occupied one of the hexagons. If an error was made during the first selection, the sixth (empty) hexagon gave the user the option to return to the first stage. Then, this two-step process was repeated to spell a complete word.

This was another gaze-independent speller, which might be appropriate for advanced-stage ALS patients. As this was an MI-based speller, which means the user has the ultimate control over the system (no external stimulation was necessary), the user had to practice using the system. As the user had more control over the system, fewer errors were expected to occur. The system’s spelling speed was also dependent to some extent on the speed at which the user was controlling the speller. All in all, this was a state-of-the-art speller system with the advantages of using MI: no necessary stimulations and gaze-independency. However, the typical disadvantages of an MI system were also present, i.e., the extended training periods, the incurred fatigue, and the increased complexity of the data analysis.

#### 4.4.2. Oct-O-Spell

Another recent MI-based speller, the Oct-O-Spell was introduced in [40]. The GUI is similar to the Hex-O-Spell. The system was controlled asynchronously by a brain switch, which means that the user can turn the speller on and off by a specific brain signal. In the first phase, the interface showed an octagon divided equally into eight sections. These sections contained in total 26 letters, six letters, digits, or symbols, each. The second phase was dependent on the first stage selection. The sector selected during the first stage was unfolded across the eight sectors (there were only six characters in each section to unfold, the commands “Back” and “Delt” were added to have eight sectors in the second stage). For only two selections from the second stage, a third stage was implemented, to verify whether the user really wants to quit (“Yes” or “No”), or, after selecting the command “F1”, to choose which symbol (“∗”, “@”, “?”, “+”, “!”, “#”) should be written. Words suggestions appeared to the user after selecting several characters, which were simply selected by entering the corresponding number. 

This interface showed a similar performance to other BCI spellers, especially hybrid BCI spellers. The interface was also tested without the predictive text. Although this case showed higher performance than the mode with the suggested words option, there was no significant difference between the two modes.

#### 4.4.3. Other MI Spellers

D’albis et al. [94] presented a novel MI-based BCI speller. The GUI consisted of four rectangles at the edges of the screen. Three boxes (upper, right, and left) contained English characters. The fourth bottom box contained commands to help the user to control the speller, such as undo, delete, switch from letters to numbers, and quit the interface. Specific imaginary movements could be used to select each of the boxes. The left and right boxes could be selected by imagining the movement of the left and right arm, respectively. The upper box was activated by the movement of both arms, and the lower box by both legs. A predictive method was applied by them to lower the number of steps necessary for selection. This was done by enabling/disabling each character according to their probability to follow the already written text. When one of the boxes was selected, the enabled letters inside were extended for a single-character selection step. 

Only three healthy subjects tested this paradigm and showed a humble performance; however, it proved the concept of the suggested interface. An additional advantage of this system was the embedded prediction module, which displayed suggested words for the user to select. 

The GUI presented in [41] (also previously mentioned in [106] as Bremen speller) is a diamond-shaped interface divided into steps. The letters are arranged according to their usage frequency and into two layers. The layer 2 contained mostly numbers, three letters with the least usage frequency, and a delete symbol. By four different imaginary movements, the user could control a cursor and change between layers. The commands “up”, “down”, “left”, “right”, and “enter” were shown on the screen to move the cursor and select the target character. 

The point of using SMR, instead of visual evoked potentials, was to avoid the uncomfortable stimulation technique. The system reported an average accuracy of 85% and proved the concept of operating the speller by using MI instead of visual stimuli. This latter point can attract more late-stages ALS patients to adopt this speller, as no eye movement was required.

### 4.5. Hybrid

To combine the advantages of two different systems, hybrid systems were developed to include more than one type of BCI or Human Machine Interface (HMI) paradigm, in general [109].

#### 4.5.1. SSVEP + P300

In [95], another approach was presented, which can be considered as a T9 speller, given that the 36 letters and numbers were divided into nine groups, requiring only nine stimulation frequencies. It was a hybrid BCI system based on SSVEP and P300, with four different characters per group, which were flickering periodically in a random order. The flickering stimulus elicited an SSVEP while the “oddball paradigm” of random characters appearing in each group was responsible for the P300. Each character appeared in different color and was placed to improve the P300 signal and the performance of the system. The hybrid system showed superior performance when compared to either the SSVEP or theP300. The individual SSVEP and P300 only systems resulted in 89% and 90% accuracies, respectively, while the accuracy of the hybrid system was 93%. As for the ITR, it was 13.0, 19.9, and 31.8 bits/min for the SSVEP, P300, and the hybrid, respectively.

#### 4.5.2. 60 Target Hybrid SSVEP/EMG (Electromyogram) 

Lin et al. [96] created a 6 × 10 speller matrix on an LCD monitor, containing 60 stimuli. The 60 characters were divided into four equal groups, resulting in 15 characters per group flickering at 15 different frequencies. To select one of the groups, the user had to make a fist movement. For each group, a specific number of fist movements were required. Group 1, 2, 3, and 4 required zero, one, two, and three movements, respectively. After selecting the desired group, the users needed to select the target letter by gazing at it to elicit an SSVEP response. 

This combination of SSVEP and EMG resulted in one of the highest mean ITR values of all the systems mentioned in this review, i.e., 90.9 bits/min, with a reasonable average accuracy of 85.80%. As neither SSVEP nor EMG requires training, the system was relatively easy to use for a wide group of users. However, as EMG requires actual physical movement (in this case, wrist movement), it limits the number of users who could profit from such a high-performing speller.

#### 4.5.3. Consonant/Vowel Lists

In [97], a hybrid BCI speller system based on Motor Evoked Potential (MEP) (a type of MI) and P300 was presented. The idea was to use the MEP when there was a low number of targets and the P300 when there were more of them. The speller consisted of two lists, one containing the consonants and the other the vowels. The letters were arranged according to their probability of usage. By imaginary left- and right-hand movements, the user chose between the lists. Once a list was selected, the letters started flickering, and the user focused on the target letter for selection. After three spelled letters, suggested words appeared on the screen and could be selected by the user. The word printed on the screen was followed by a space. The system showed faster performance but reduced accuracy. 

The reported results showed average ITR and accuracy levels compared to other studies mentioned in this review. However, the idea of dividing the letters into consonant and vowel lists might make it easier for users to spell and can achieve higher typing speed after a number of trials. It was also proven that the hybrid system achieved better performance than the individual P300 or MI. This interface might be helpful for users who have difficulties focusing on the flashing Matrix speller. However, this speller was only tested by two healthy participants.

#### 4.5.4. MI + P300

In [42], a self-paced hybrid BCI speller was introduced. It was a matrix speller based on Farwell and Donchin’s speller, but it was controlled by an MI switch. A matrix with the size of 6 × 7, including 26 English letters, 10 numbers, comma “,”, dot “.”, “Del”, and “Exit”, was initially turned OFF at the start of the experiment. The user had to intentionally change his/her state of mind by MI to turn the system ON and start the flashing of the stimuli. The hybrid speller showed exciting results, the average classification accuracy and the ITR were 92.93%, and 41.23 bits/min, respectively. These results are relatively within the range of the commonly tested P300 matrix spellers, but, for this experiment, the user could control when to start and when to stop spelling intentionally, without affecting the performance of the speller.

## 5. Discussion

In addition to the developments presented in this review paper, various modalities have been presented throughout the years with the primary aim to improve the quality of life of users with disabilities. Eye tracking devices are a successful example, which are commercially available off the shelf for users and also used for further development of new applications for MND patients [110]. Many studies have been carried out to compare and/or combine BCI systems with eye trackers. One of the most recent studies is our study [111], which merges both systems to develop a hybrid speller combining the advantages of both systems in a competent speller. More information about eye tracking and the comparison between eye tracker spellers and BCIs can be found in [111]. 

Other systems have a special chin joystick, which could assist the user to manipulate an assistive robot, as presented in [112]. To mention other control modes, in [113], computer and assistive devices were controlled by the tongue, and, in [114], infrared sensors were used to detect head movement to control a computer mouse. All these methodologies are very useful and can benefit many people with disabilities. Although some of these methods can be faster and more accurate than BCI, when applied to spelling applications, they restrict the number of potential users, as they are only beneficial for patients who still maintain some motor control. BCIs usually require minimal or no muscular movement, qualifying BCI systems to be suitable for a wider group of users. 

Spelling applications are our key focus of this review. Nevertheless, it is worth mentioning that BCIs are applied for a lot of other purposes, e.g., robot control [115,116,117], wheelchair control [118,119], web browsing [120], general control of an operating system with a virtual keyboard, as presented in [121], gaming [122], every-day electronic device control [123,124,125], and ADHD attention training [126], just to mention a few. A lot of these applications are also useful for people with neuromuscular malfunctions, and a number of them can be suitable for healthy users.

Although BCIs have a lot of advantages and benefits, there is room for improvements. Usually, BCI systems require some time and help to be set up. It is tricky for a person with no BCI experience to set up a commonly used BCI system. One of the reasons is the setup of the electrodes. The used electrodes need to be fixed at specific positions, and it is also essential to apply electrolyte gel correctly for EEG-based measurement. As an example, in [127,128], other types of electrodes and setup methods were proposed. Another challenge is the portability. Researchers have been working lately to overcome this barrier to achieve a more portable BCI system; just to mention some examples: [128,129,130,131].

In addition to working on the above-stated challenges, researchers always aim for BCI systems with better performance. Here, we discuss the developments made towards this goal in the aspect of GUI changes. Many articles have been published with respect to other properties, especially data processing and analysis of a BCI. Much more papers were presented in this field (examples: [132,133,134,135]).

Back to the main topic of discussion, the BCI spellers, various interfaces and systems are reported in various literature libraries. As each presented system had its own variables, parameters, and conditions, it is not possible to carry out an objective comparison between different GUI spellers. However, we can still discuss some advantages and disadvantages of the systems described in the previous section of this review. Table 2, Table 3 and Table 4 summarize the studies mentioned above, while stating the main specifications of each system, such as accuracy, information transfer rate (ITR), and the number and type of subjects who participated in the studies. Table 2 includes all the variations and studies which were built on the original Matrix Speller. Table 3 presents a summary of the performance of other P300-based spellers which were not directly inspired by the Matrix Speller, and, finally, Table 4 presents the performance of the above-mentioned SSVEP-based spellers, MI spellers, and hybrid spellers. We can see from these tables that most of the developments made during the decade are based on P300 BCIs, especially on the Matrix speller GUI.

Another reason why the performance varies from one study to another is the use of different resources. Different teams utilize different hardware. There are several available bioamplifiers on the market as well as a variety of EEG caps and electrodes. Additionally, researchers usually build the software which is suitable for them. Some develop their own software and others would make use of available tools on the market. These parameters affect the performance greatly. A first step to conduct a subjective comparison between different BCI systems is to make sure that the same hardware and software are being implemented.

It is also noticeable from the tables that there are performance variations between different systems in the same category. A main reason behind this is that the ITR and accuracy are calculated differently for different interfaces, which means that it is almost impossible to carry out an objective evaluation or comparison between the systems. In some cases, it is obvious that the improvement in the spelling speed affected the accuracy negatively or vice versa. For example, the Center Speller or the RSVP spellers (see Table 2 for more details) were specifically designed to achieve high accuracy and gaze-independent spelling. They successfully achieved their goals; however, the typing speed was affected significantly.

In other cases, a typing mistake requires time for correction, affecting the spelling speed. To eliminate this negative effects, researchers integrated Error-related Potentials (ErrPs) into BCI spellers. The ErrP signal is generated 50–100 ms after an error is detected by the user. The error might be due to a human error from the user’s side, or it can be that the machine behaved differently from what the user expected. In [84,136,137], the ErrP was used to automatically detect and delete the errors in a BCI speller. The merging of ErrP with P300 aimed to increase the accuracy of the speller, while avoiding affecting the spelling speed, as the correction was done automatically. In our opinion, accuracy is more important for the subjects. During many experiments, we observed that the subjects were much more frustrated by typing mistakes than by a slow-performing system.

P300 is very popular among BCI researchers because of its relatively high ITR and the minimal user training required (compared to MI). However, in general, P300 spellers have several disadvantages. As the number of commands increases in a P300 speller, the number of trials increases as well, leading to a slower performance. As the feature extraction mostly depends on identifying the point of intersection of which row and column elicited the signal, at least two flashes are required for each target, which, again, increases the classification times. Although scientists have been working lately to develop gaze-independent P300 spellers, most of these systems require visual attention or even gaze shifting. The gaze shift dependency might not be applicable for patients with severe paralysis. It is worth noting that the modifications developed and applied to the original Matrix Speller paradigm showed better performance than that of the traditional RCP, especially when tested with MND patients. Some researchers worked on improving the performance of P300 spellers by developing classification algorithms to build an asynchronous P300-based BCI. As an example, the team in [138] combined a P300 speller similar to the Geospell (already mentioned in Section 4.2.4), which was gaze-independent, with an asynchronous algorithm. However, these results were not significantly different. Later, in [139], the group achieved promising results by embedding a self-calibration module to the system. This improvement included an algorithm which automatically recalibrated the parameters of the classifier and adjusted them according to the personal performance of the user. Accordingly, the system could adjust the parameters to attain the optimum accuracy and typing speed.

Some of these challenges are overcome in other BCI paradigms. For example, SSVEP does not require a minimum number of flashes to elicit a response. Thus, it can be used to achieve faster spellers. It also requires no training at all on the user’s side. On the other hand, it has been observed that some participants have a low SSVEP response, which is almost impossible to detect and use as a control signal when a high number of stimuli is presented. As for MI, once the user receives the required training, the system can achieve impressive results. However, the training might take a long time. 

The recently developed mVEP paradigm overcomes several other challenges which were faced during the development of other BCI systems. It is elicited entirely by the motion and the motion behavior of the visual stimulus. Thus, such system is not sensitive to contrast, illumination, color, or size of the stimulus. An mVEP-based paradigm does not require any previous training as well. 

Another approach to avoid the gaze dependency problem is to utilize other sensory modalities than vision. As mentioned in the introduction, auditory and tactile ERP researches have been published with promising results. In [140], the Farwell and Donchin P300 speller was modified into a visual–auditory speller, which showed similar performance compared to the visual paradigm. In [141], an auditory matrix speller was presented, where the authors used natural animal sounds as stimuli. The system was tested by impaired subjects and resulted in a relatively high performance. However, these systems still require a lot of training and familiarization. 

An example of a tactile ERP system is the Braille-like system developed in [142] as a P300-based tactile BCI system. Two or four different types of tactile stimuli were assigned to intentions, such as “yes” and “no”, “right” and “left”, or “up” and “down.” The stimulation was a tactile stimulator using a piezoelectric actuator, used with the braille system, consisting of eight cylindrical pins which could be pressed lightly against the fingertips of the user. The number and the positions of the pins moving up defined the stimulus. Another recent study [143] combined tactile and auditory stimuli to form a hybrid BCI system. Although the performance of BCI spellers based on tactile and/or auditory stimulus (a stimulus other than vision) is still not better than the visual stimuli-based BCI spellers, specifically when used with healthy participants, they are of great importance when considering patients with eyesight problems or in late stages of ALS.

It is evident from Table 2, Table 3 and Table 4, that most of the studies were conducted using the P300 paradigm. This can be due to its popularity, its many advantages, and the fact that it has been studied for many years. This can guide us to deduce that there are more development opportunities in the other BCI paradigms. Overall, researchers have been working for many years to develop efficient BCI communication applications, which are safe, affordable, reliable, easy to setup, easy to use, and achieving a fast communication speed. From what we discussed above, BCI spellers can be a suitable option for people who have no other means of communication with their surroundings. However, BCI spellers are not fast enough, compared to other regular communication methods, like typing or speaking, neither as fast as other systems, like spellers based on eye trackers. Also, BCIs are relatively complicated to set up and require specific skills. A typical BCI system is not considered to be very portable. Some of the spellers discussed above aimed to tackle these challenges and gaps, but still more developments are required. Combining different BCI systems or combining BCI with other non-BCI systems can also lead to promising results. As already discussed, some spellers merged P300 with SSVEP, and some others combined them with other systems, like eye trackers. The results achieved by the hybrid systems were usually faster and/or more accurate. The general aim is to achieve a BCI speller which is as fast as other communication methods, easy to carry around and set up, comfortable to use for short terms and on the long run, and suitable for the broadest range of users. 

## 6. Conclusions

All the systems discussed above were studied and presented with the aim to improve BCI spellers. Throughout the years, scientists have worked on spelling systems to make them faster, more accurate, more user-friendly, and, most of all, able to compete with traditional communication methods. On the other hand, a lot of gaps are still to be closed to achieve efficient BCI spellers. More emphasis needs to be given to GUI design to satisfy the needs of the end-users. In addition, more testing with patients is required. From the summary tables, we can see that only five systems were tested by afflicted subjects. BCI spellers provide a practical and efficient way for people who cannot communicate through traditional methods to be able to participate in their social lives and careers. The different GUIs described and discussed during this review, as well as the other different systems mentioned, may provide an inspiring starting point for further studies and improvements.

## Figures and Tables

**Table 1:** BCI spellers’ taxonomy. The table also classifies the papers presented in this review according to the suggested taxonomy.

*BCI Paradigm*		P300 45 Studies 65% of Total	SSVEP 16 Studies 23% of Total	MI 4 Studies 6% of Total	Hybrid 4 Studies 6% of Total
**Operation Modality**	Asynchronous	0.0%	68.8%	75.0%	25.0%
	21.7%		[28,29,30,31,32,33,34,35,36,37,38]	[39,40,41]	[42]
	Synchronous	100.0%	31.3%	25.0%	75.0%
	78.3%	[7,43,44,45,46,47,48,49,50,51,52,53,54,55,56,57,58,59,60,61,62,63,64,65,66,67,68,69,70,71,72,73,74,75,76,77,78,79,80,81,82,83,84,85,86,87,88]	[89,90,91,92,93]	[94]	[95,96,97]
**Gaze Dependency**	Gaze Independent	15.6%	6.3%	75.0%	0.0%
	15.9%	[74,78,79,80,82,83,84]	[37]	[39,40,94]	
	Gaze Dependent	84.4%	93.8%	25.0%	100.0%
	84.1%	[7,43,44,45,46,47,48,49,50,51,52,53,54,56,57,58,59,60,62,63,64,65,66,67,68,69,70,71,72,73,75,76,77,81,85,86,87,88]	[28,29,30,31,32,33,34,35,36,38,89,90,91,92,93]	[41]	[42,95,96,97]
**Selection Modality**	Direct Target Selection	100.0%	87.5%	25.0%	100.0%
	92.8%	[7,43,44,45,46,47,48,49,50,51,52,53,54,55,56,57,58,59,60,61,62,63,64,65,66,67,68,69,70,71,72,73,74,75,76,77,78,79,80,81,82,83,84,85,86,87,88]	[29,30,31,32,33,34,35,36,38,89,90,91,92,93,98]	[94]	[42,95,96,97]
	Moving Cursor	0.0%	12.5%	75.0%	0.0%
	7.2%		[28,37]	[3,4,5,6,7,8,9,10,11,12,13,14,15,16,17,18,19,20,21,22,23,24,25,26,27,28,29,30,31,32,33,34,35,36,37,38,39,40,41]	
**Stimuli Modality**	Constant Flashing	0.0%	100.0%	0.0%	50.0%
	26.1%		[28,29,30,31,32,33,34,35,36,37,38,89,90,91,92,93]		[95,96]
	Periodic Flashing	82.2%	0.0%	0.0%	75.0%
	58.0%	[7,43,44,46,47,48,49,57,58,59,60,62,63,64,65,66,67,68,69,70,71,72,73,74,75,76,77,78,79,80,81,83,84,85,86,87,88]			[42,95,97]
	Moving/Animation	17.8%	6.3%	0.0%	0.0%
	13.0%	[45,50,51,52,53,54,56,82]	[93]		
	No visual Stimuli	0.0%	0.0%	100.0%	0.0%
	5.8%			[39,40,41,94]	
**Word Prediction**	Yes	13.3%	18.8%	25.0%	0.0%
	14.5%	[62,63,64,65,75,76]	[28,37,38]	[94]	
	No	86.7%	81.3%	75.0%	100.0%
	85.5%	[7,43,44,45,46,47,48,49,50,51,52,53,54,55,56,57,58,59,60,66,67,68,69,70,71,72,73,74,77,78,79,80,81,82,83,84,85,86,87,88]	[29,30,31,32,33,34,35,36,89,90,91,92,93]	[39,40,41]	[42,95,96,97]

**Table 2:** Summary of all spellers discussed in this review which are based on the P300 Matrix Speller.

Topic/Speller Name	Reference		Subjects	Mean ITR/Typing Speed	Mean Accuracy
**Matrix Speller**	[7]	Farwell and Donchin 1988	4 healthy	12 bits/min	95.0%
**Stimuli Variations**	[43]	Yeom et al. 2014a	4 healthy	66.3 bits/min	64.7%
	[44]	Obeidat et al. 2015	14 healthy	13.7 bits/min	93.3%
	[45]	Liu et al. 2010	4 healthy	rotation stimuli: 35.8 bits/min	rotation stimuli: 89.06%
	[46]	Shi et al. 2012	7 healthy	SBP433: 26.8 bits/min	99.7%
	[47]	Eom et al. 2013	5 healthy	13.5 s/char	79.2%
	[48]	Jin et al. 2010	8 healthy	14.8 bits/min	92.9%
	[49]	Polprasert et al. 2013	10 healthy	23.82 bits/min	84.0%
**Familiar Faces and symbols**	[50]	Kaufmann et al. 2011	20 healthy	N/A	Max 100%
	[51]	Li et al. 2015a	17 healthy	N/A	N/A
	[52]	Li et al. 2015b	12 healthy	39.0 bits/min	86.1%
	[53]	Kaufmann and Kübler 2014	8 healthy	~80 bits/min	81.25%
	[54]	Yeom et al. 2014b	15 healthy	RASP-F: 53.7 bits/min RASP: 32.8 bits/min	84.0% 90.7%
	[56]	Kathner et al. 2015	18 healthy + 1 LIS	15.5–16.2 bits/min	94–96%
**Variation of letters arrangement**	[57]	Ahi et al. 2011	14 healthy	55.32 bits/min	87.14%
	[58]	Li et al. 2011	10 healthy + 10 NMD	N/A	79.7–28.7%
	[59]	Jin et al. 2012	9 healthy	18-P: 29.9 bits/min 21-P: 27.1 bits/min	18-P: 93.3% 21-P: 94.8%
	[60]	Sakai and Yagi 2011	9 healthy	N/A	N/A
**Matrix Speller with Prediction**	[62]	Ryan et al. 2011	24 healthy	17.71 bits/min	84.88%
	[63]	Kaufmann et al. 2012	20 healthy	Max 25 bits/min	>70%
	[64]	Akram et al. 2013	4 healthy	26.1 s/char	77.5%
	[65]	Akram et al. 2014	10 healthy	26.13 s/char	77.14%
**Other languages**	[66]	Minett et al. 2010	30 healthy	14.5 bits/min	>60%
	[67]	Minett et al. 2012	24 healthy	4.23 bits/min	82.8%
	[68]	Yu et al. 2016	10 healthy	39.2 bits/min	92.6%
	[69]	Kabbara et al. 2015	11 healthy	N/A	88–95%
	[70]	Lee et al. 2011	3 healthy	N/A	100% after training
	[71]	Yamamoto et al. 2014	4 healthy	N/A	93%
	[72]	Ikegami et al. 2014	7 ALS patients + 7 healthy	N/A	ALS: 24%, 55% healthy: 55%, 83%
**3D Blocks Matrix Speller**	[73]	Noorzadeh et al. 2014	16 healthy	N/A	~90% with 5 repetitions

**Table 3:** Summary of all other P300-based spellers which are not directly related to the Matrix Speller.

Topic/Speller Name	Reference		Subjects	Mean ITR/Typing Speed	Mean Accuracy
**Chroma Speller**	[74]	Acqualagna et al. 2013	9 healthy	1.4 char/min	88.4%
**T9**	[76]	Ron-Angevin et al. 2015	11 healthy + 1 with ALS	N/A	N/A
	[75]	Akram et al. 2015	10 healthy	26.125 s/char	N/A
**Checkerboard Paradigm**	[77]	Postelnicu and Talaba 2013	10 healthy	21.74 bits/min	90.63%
**Geospell**	[79]	Liu et al. 2011	8 healthy	1.38 char/min	RP: 87.8% FP: 84.1%
	[78]	Aloise et al. 2012	10 healthy	1.86 char/min	78%
	[82]	Zhou et al. 2016	10 healthy	N/A	N/A
**GIBS**	[80]	Pires et al. 2011	4 healthy	16.67 bits/min	96.02%
**LSC Speller**	[81]	Pires et al. 2012	10 healthy + 7 ALS + 5CP + 1 DMD + 1 SCI	26.11 bits/min	89.9%
**Hex-O-Spell with ERP**	[83]	Treder et al. 2011	13 healthy	2 char/min	Hex-O-Spell: 90.4% Cake Speller: 88.0% Center Speller: 97.0%
	[84]	Schmidt et al. 2012	11 healthy	2.75 char/min	89.1%
**Rapid serial visual presentation RSVP**	[87]	Acqualagna and Blankertz 2013	12 healthy	1.43 char/min	94.8%
	[85]	Acqualagna et al. 2010	9 healthy	N/A	90%
	[86]	Acqualagna and Blankertz 2011	12 healthy	2 char/min	94.8%
	[88]	Sato and Washizawa 2016	11 healthy	2 × 2: 0.70 bits/s 2 × 3: 0.85 bits/s	2 × 2: 74.4% 2 × 3:70.3%

**Table 4:** Summary of the spellers discussed in this review which are based on SSVEP, MI, and Hybrid system.

Topic/Speller Name	Reference		Subjects	Mean ITR/Typing Speed	Mean Accuracy
**Bremen Speller**	[28]	Volosyak et al. 2011	7 healthy	32.71 bits/min	Correct spelling only
**Multi-Phase Spellers**	[29]	Volosyak et al. 2017	20 healthy	group A: 27.36 bits/min group B: 16.10 bits/min	group A: 98.49% group B: 91.13%
	[30]	Cecotti 2010	8 healthy	37.62 bits/min	92.25%
	[31]	Cao et al. 2011	4 healthy	61.64 bits/min	98.78%
	[32]	Ansari and Singla 2016	20 healthy	13 chars/min	96.04%
**Multi-Target One-Phase Spellers**	[33]	Wang et al. 2010	3 healthy	75.4 bits/min	97.2%
	[34]	Chen et al. 2015	12 healthy	4.45 bits/min	91.04%
	[89]	Nakanishi et al. 2018	20 healthy	325.33 bits/min	89.83%
	[35]	Spüler et al. 2012	9 healthy	143.95 bits/min	96.18%
	[36]	Wei et al. 2017	4 healthy	129.58 bits/min	90.5%
**RC SSVEP Speller**	[90]	Yin et al. 2015b	11 healthy	41.08 bits/min	~95%
	[91]	Yin et al. 2013	12 healthy	56.44 bits/min	93.85%
	[92]	Yin et al. 2014	14 healthy	RC: 53.06 bits/min SL: 44.7 bits/min	N/A
	[93]	Yin et al. 2015a	13 healthy	50.41 bits/min	95.18%
**Flash-Type Speller**	[37]	Nezamfar et al. 2016	3 healthy	6.2–11 s/char	95.5–97%
**DTU BCI Speller**	[38]	Vilic et al. 2013	9 healthy	21.94 bits/min	90.81%
**Hex-O-Spell**	[39]	Blankertz et al. 2006	2 healthy	max 7.6 char/min	error free measurements
**Oct-O-Spell**	[40]	Cao et al. 2017	3 healthy	Non-PTE: 69.16 bits/min PTE: 62.39 bits/min	Non-PTE: 98.3% PTE: 96.6%
**Other MI Speller**	[94]	D’Albis et al. 2012	3 healthy	max 3 char/min	average N/A
	[41]	Jingwei et al. 2011	5 healthy	N/A	85.0%
**SSVEP+P300**	[95]	Chang et al. 2016	10 healthy	31.8 bits/min	93%
**SSVEP+EMG**	[96]	Lin et al. 2016	10 healthy	90.9 bits/min	85.8%
**Consonant/Vowels list**	[97]	Roula et al. 2012	2 healthy	11 s/char	70%
**MI+P300**	[42]	Yu et al. 2016	11 healthy	41.23 bits/min	92.93%
